# Isolation and Identification of *Burkholderia stagnalis* YJ-2 from the Rhizosphere Soil of *Woodsia ilvensis* to Explore Its Potential as a Biocontrol Agent Against Plant Fungal Diseases

**DOI:** 10.3390/microorganisms13061289

**Published:** 2025-05-31

**Authors:** Xufei Zhu, Wanqing Ning, Wei Xiao, Zhaoren Wang, Shengli Li, Jinlong Zhang, Min Ren, Chengnan Xu, Bo Liu, Yanfeng Wang, Juanli Cheng, Jinshui Lin

**Affiliations:** 1 Shaanxi Key Laboratory of Research and Utilization of Resource Plants on the Loess Plateau, College of Life Sciences, Yan’an University, Yan’an 716000, China; 17806813772@163.com (X.Z.); 18387120837@163.com (W.N.); 18792388186@163.com (W.X.); zhaorenwang00@163.com (Z.W.); 18181017935@163.com (S.L.); zhangjinlong04@yau.edu.cn (J.Z.); minren_edu@163.com (M.R.); xuchengnan1981@sina.com (C.X.); liubo4552@126.com (B.L.); wyfcollege@sohu.com (Y.W.); 2 State Key Laboratory for Crop Stress Resistance and High-Efficiency Production, Northwest A&F University, Xianyang 712100, China

**Keywords:** *Burkholderia stagnalis*, genome sequencing, biological control, colonization

## Abstract

Plant fungal diseases remain a major threat to global agricultural production, necessitating eco-friendly and sustainable strategies. Conventional chemical fungicides often lead to the development of resistant pathogen strains and cause environmental contamination. Therefore, the development of biocontrol agents is particularly important. In this study, we identified *Burkholderia stagnalis* YJ-2 from the rhizosphere soil of *Woodsia ilvensis* as a promising biocontrol strain using 16S rRNA and whole-genome sequencing. This strain demonstrated broad-spectrum antifungal activity against plant fungal pathogens, with its bioactive extracts maintaining high stability across a temperature range of 25–100 °C and pH range of 2–12. We used in vitro assays to further show that the metabolites of *B. stagnalis* YJ-2 disrupted the hyphal morphology of *Valsa mali*, resulting in swelling, reduced branching, and increased pigmentation. Fluorescence labeling confirmed that *B. stagnalis* YJ-2 stably colonized the roots and stems of tomato and wheat plants. Furthermore, various formulations of microbial agents based on *B. stagnalis* YJ-2 were evaluated for their efficacy against plant pathogens. The seed-coating formulation notably protected tomato seedlings from *Alternaria solani* infection without affecting germination (*p* > 0.1), while the wettable powder exhibited significant control effects on early blight in tomatoes, with the preventive treatment showing better efficacy than the therapeutic treatment. Additionally, the *B. stagnalis* YJ-2 bone glue agent showed a substantial inhibitory effect on apple tree canker. Whole-genome analysis of *B. stagnalis* YJ-2 revealed a 7,705,355 bp genome (67.68% GC content) with 6858 coding genes and 20 secondary metabolite clusters, including three clusters (YJ-2_GM002015-YJ-2_GM002048, YJ-2_GM0020090-YJ-2_GM002133, and YJ-2_GM06534-YJ-2_GM006569) that are related to the antifungal activity of YJ-2 and are homologous to the biosynthetic gene clusters of known secondary metabolites, such as icosalide, ornibactin, and sinapigladioside. We further knocked out core biosynthetic genes of two secondary metabolic gene clusters and found that only the YJ-2_GM006534-YJ-2_GM006569 gene cluster had a corresponding function in two potential antifungal gene clusters. In contrast to the wild-type strain YJ-2, only deletion of the YJ-2_GM006563 gene reduced the antifungal activity of *B. stagnalis* YJ-2 by 8.79%. These findings highlight the biocontrol potential of *B. stagnalis* YJ-2, supporting a theoretical foundation for its development as a biocontrol agent against plant fungal diseases and thereby promoting sustainable agricultural disease management.

## 1. Introduction

Fungal diseases are responsible for up to 42% of global crop losses [1]. Although current strategies such as resistant cultivar breeding, crop rotation, and chemical fungicides are widely used, their effectiveness is increasingly constrained by genetic limitations, complex field conditions, and rising pathogen resistance [2]. Traditional chemical control can inhibit the spread of fungal diseases in the short term, but long-term use has led to plant fungi developing resistance to fungicides [3]. Additionally, pesticide infiltration disrupts the soil microbiome, exacerbating the degradation of farmland ecosystems. Prolonged chemical use also leads to pesticide residues in fruits, as well as environmental pollution in orchards [4]. Growing environmental concerns and higher food quality standards have attracted the attention of researchers to biological control for its safety, non-toxicity, and low resistance risk [5,6].

Biological control is a control method that uses beneficial microorganisms and their metabolites to inhibit or kill harmful species [7]. In essence, it aims to decrease the occurrence of plant diseases by employing natural mechanisms, such as competition and bacteriolysis, to achieve effective prevention and control of plant infection [8,9]. Currently, the biological control of plant fungal diseases primarily includes the use of beneficial microorganisms, including fungi, actinomycetes, and bacteria, and their secondary metabolites, antibacterial extracts, and botanical pesticides [10,11,12,13,14]. Various bacteria have become important tools in the biological control of plant diseases due to their unique and diverse antimicrobial mechanisms and strong environmental adaptability [15]. Currently, common biocontrol bacteria are mostly found in the *Bacillus*, *Pseudomonas*, *Streptomyces*, and *Burkholderia* genera [16].

*Burkholderia* species have gradually become a research hotspot due to their unique biological characteristics, widespread geographic distribution, and significant antibacterial effects, attracting many researchers to investigate their potential in controlling plant fungal diseases [17]. For example, *Burkholderia* sp. ESS4, isolated from the roots and stems of sugarcane in Brazil, produces the antifungal metabolite pyrrolnitrin [18]. Similarly, the seed-coating agent composed of *Burkholderia cepacia* MCI7 was shown to prevent the infection of major fungal pathogens, such as *Fusarium moniliforme*, in corn [19]. Additionally, *Burkholderia ambifaria* BC-F inhibited the growth of hyphae from various pathogenic fungi by releasing bacteriostatic compounds. The seed-coating agent made of *B. ambifaria* BC-F significantly reduced the infection of *Pythiumultimum* on cucumber and soybean, thereby promoting healthy plant growth [20]. Another example is the antifungal compound CF66I produced by *B. cepacia,* which suppresses the growth of many pathogenic fungi [21]. At high doses, CF66I disrupts the cell membrane and kills the pathogen, while at low doses, CF66I primarily inhibits the pathogen by causing fungal hypha swelling and altering the structure of the cell wall [22]. Furthermore, Sandania et al. [23] discovered that *Burkholderia arboris* F35, *Burkholderia gladioli* F79, and *Burkholderia rinojensis* F80 displayed an antifungal effect on *Colletotrichum truncatum*. The inhibition rate of *B. gladioli* F79 and *B. rinojensis* F80 on the spore germination of *C. truncatum* was nearly 100% in vitro, and microscopic observation revealed that *C. truncatum* hyphae were thickened, swollen, and deformed by *B. rinojensis* F80. In vivo experiments also demonstrated that inoculating pepper seeds with *B. arboris* F35, *B. gladioli* F79, and *B. rinojensis* F80 significantly reduced the rate of *C. truncatum* infection. Additionally, *B. Arboris* F35, *B. gladioli* F79, and *B. rinojensis* F80 exhibited 100% control over anthracnose during the color-breaking stage. Taken together, *Burkholderia* sp. shows significant potential in controlling plant fungal diseases [24].

In this study, *B. stagnalis* YJ-2 was isolated from the rhizosphere soil of *Woodsia ilvensis*. To study the biocontrol mechanism of *B. stagnalis* YJ-2, various formulations of microbial agents, including a seed-coating agent, wettable powder, and bone glue agent, were developed for different plant fungal diseases, and their biocontrol effects were explored. We also constructed *B. stagnalis* YJ-2 (pBBR-*gfp*-mut3), which carries a green fluorescent protein (GFP) gene, and the colonization of this strain in plants was analyzed using potted wheat and tomatoes as plant models. Furthermore, whole-genome sequencing of *B. stagnalis* YJ-2 was completed to analyze and predict the secondary metabolite synthesis gene clusters, which allowed the identification of functional genes regulating the antifungal activities of *B. stagnalis* YJ-2. This study not only provides a theoretical basis for studying the antifungal mechanism underlying *B. stagnalis* YJ-2, but it also demonstrates the potential of *B. stagnalis* YJ-2 as a biological control agent against plant fungal diseases. Thus, *B. stagnalis* YJ-2 is an alternative high-quality biological resource for the control of plant fungal diseases and the promotion of sustainable agricultural disease management.

## 2. Materials and Methods

### 2.1. Samples and Test Strains

Soil samples for rhizosphere bacteria isolation were taken from the rhizosphere of *W. ilvensis* in Tianhe District, Guangzhou City, Guangdong Province, China. The five tested plant pathogenic fungi—*Valsa mali* strain ACCC39338, *Bipolaris sorokinianum* strain ACCC36341, *Fusarium graminearum* strain ACCC39334, *Exserohilum turcicum* strain ACCC39449, and *Alternaria solani* strain ACCC36023—were provided by Yan’an University Disease Green Prevention and Control Laboratory (Yan’an City, Shaanxi Province, China) and preserved by the Agricultural Culture Collection of China.

### 2.2. Isolation and Screening of Bacterial Strains

Bacteria were isolated as previously described [25]. Briefly, the soil sample (1 g) was added to distilled water (90 mL) in a 250 mL conical bottle and incubated for 30 min on a rotating shaker at 37 °C at 180 rpm. The sample was then diluted to 10^−6^, and 0.1 mL of the 10^−6^ dilution was then plated on *Pseudomonas* isolation agar (PIA) [26] and incubated at 37 °C for 3 days. A single colony was then selected and streaked onto PIA until a pure culture was obtained. Using *V. mali* as an indicator strain, the bacterial strain exhibiting the best antifungal activity was screened using the plate confrontation method [27].

### 2.3. Fermentation Culture of Strain YJ-2 and Preparation of the Active Extract

The fermentation culture of strain YJ-2 was performed as previously reported [28]. Briefly, strain YJ-2 was inoculated into tryptic soy broth (TSB) [29] culture medium and cultured at 37 °C with shaking at 200 rpm for 1 day. The obtained YJ-2 seed liquid was passaged 1:100 in yeast sucrose peptone (YSP) [30] liquid culture medium and then cultured at 37 °C with shaking at 200 rpm for 3 days to obtain the strain YJ-2 fermentation broth. The fermentation broth of strain YJ-2 was centrifuged at 5702× *g* for 10 min, and both the fermentation supernatant and cell pellet were collected. After filtering through a 0.22 µm sterile filter membrane, two times the volume of acetone was added, and the cell-free supernatant extract was obtained by concentrating it in a rotary evaporator. YJ-2 cell fluid was soaked with acetone for 1 day with ultrasonic-assisted extraction. The acetone extract was collected by centrifugation at 12,830× *g* for 5 min and repeated three times until the acetone extract became colorless and transparent. The acetone extract was then combined, and the cell extract was acquired by concentrating the sample in a rotary evaporator. The cell-free supernatant extract and cell extract of strain YJ-2 were combined and stored at 4 °C.

### 2.4. Morphological Observation

Using criteria drawn from Bergey’s Manual^®^ of Systematic Bacteriology [31], strain YJ-2 was cultured on PIA plates, TSB plates, and YSP plates at a constant temperature of 37 °C for 1–2 days prior to observing and recording the colony morphology and the presence or absence of soluble pigments. In addition, the morphological characteristics of YJ-2 were assessed via Gram staining. The bacteria were observed under both an optical microscope (Chongqing Aopu Optoelectronic Technology Co., Ltd., Chongqing, China, UN102i) and a scanning electron microscope (Hitachi sU3800, Hitachi Scientific Instruments (Beijing) Co., Ltd., Beijing, China).

### 2.5. Biological Characteristics of Strain YJ-2

The ability of YJ-2 to produce indole-3-acetic acid (IAA) was quantitatively determined using a colorimetric method [32], where a red solution indicates the ability of the bacteria to produce IAA [33]. The 12 h culture of YJ-2 was subcultured at a ratio of 1:100 into TSB medium containing tryptophan (final concentration of 10 mg/mL) and cultured at 37 °C with shaking at 200 rpm for 1 day. The supernatant was collected by centrifugation at 5702× *g* for 10 min and mixed with Salkowski’s reagent [34] at a 1:2 ratio, using tryptophan-supplemented medium as a negative control, and then incubated for 30 min at 25 °C in the dark. The absorbance of the mixed solution was measured at 530 nm (OD _530nm_) to detect the IAA yield of strain YJ-2. In the same manner as above, a standard curve with different concentrations of IAA dilutions (0 mg/mL, 10 mg/mL, 20 mg/mL, 30 mg/mL, 40 mg/mL, 50 mg/mL, and 60 mg/mL) was prepared in a manner similar to the experimental solution.

The production of siderophores was tested with Chromeazurol S (CAS) medium [35]. A 5 µL aliquot of an overnight YJ-2 culture was inoculated in the center of the CAS plate [36]. The ability of strain YJ-2 to produce siderophores was evaluated by observing the orange circles produced around the YJ-2 bacterial lawn on the CAS plate.

YJ-2 was also inoculated onto Ashby’s Glucose Agar [37] to determine its nitrogen-fixing ability. YJ-2 single colonies were streaked on Ashby’s Glucose Agar and cultured at 37 °C for three consecutive passages to assess the nitrogen-fixation capacity of strain YJ-2. All of the experiments were performed three times.

### 2.6. Phylogenetic Identification of Strain YJ-2 Based on 16S rRNA Sequencing and Genome Sequencing

The identification of strain YJ-2 was performed using 16S rRNA and genome sequencing. The 16S rRNA gene was amplified with the primers 27 F (5′-AGAGTTTGATTGATCCTGGCT-CAG-3′) and 1492 R (5′-GGTTACCTTGTTACGACTT-3′), which are specific for bacterial 16S rRNA. The final reaction volume was 50 µL, which included 1 µL of Trans Taq-T DNA polymerase, 2 µL each of the forward and reverse primers, 5 µL of DNA template, 4 µL of dNTPs (2.5 mmol/L), 5 µL of 10× Trans Taq-T buffer, and 31 µL of ddH_2_O. The conditions for the PCR amplification were as follows: 95 °C for 5 min for 1 cycle; followed by 31 cycles of 95 °C for 50 s, 55 °C for 1 min, and 72 °C for 90 s; and finally, 72 °C for 10 min. After PCR amplification, the PCR products were analyzed by electrophoresis using a 1% agarose gel. The expected PCR product (approximately 1500 bp) was purified and cloned into vector pMD19-T, and the resulting plasmid was transformed into *E. coli* TG1 competent cells. Positive transformants were picked and sent to Beijing AugCT Biotechnology Co., Ltd., Beijing, China, for sequencing. The online blast program (https://blast.ncbi.nlm.nih.gov/Blast.cgi, accessed on 15 October 2024) was used to search for DNA sequence homology. A phylogenetic tree for YJ-2 based on the 16S rRNA sequencing was constructed using the Mega 5.0 software package as previously described [38,39]. Strain YJ-2 was inoculated in TSB medium and cultured at 37 °C with shaking at 180 rpm for 24 h. The bacteria were collected by centrifugation at 3000× *g* for 5 min and then sent to Beijing Novogene Bioinformatics Technology Co., Ltd., Beijing, China for sequencing. Genome assembly of reads was performed using the Canu software (version 2.0) based on the sequencing results. The Type Strain Genome Server (dsmz.de) (TYGS) online tool (https://tygs.dsmz.de/, accessed on 28 October 2024) and DNA hybridization method [40,41] were used to annotate the species classification and construct the species developmental tree using the Interactive Tree of Life (iTOL) online tool (https://itol.embl.de/, accessed on 30 October 2024).

### 2.7. Determination of the Bacteriostatic Spectrum

The plate confrontation assay was performed as described previously, with some modifications [27]. In brief, the pathogenetic fungi, including *V. mali*, *A. solani*, *F. graminearum*, *B. sorokinianum*, and *E. turcicum*, were cultivated on potato dextrose agar (PDA) [42] at 25 °C for 4–6 days. A pathogen disk with an approximate diameter of 5 mm was then transferred to the center of the PDA plate. The active extract of strain YJ-2 was prepared by collecting the supernatant from a 16-h culture of YJ-2 (OD₆₀₀ ≈ 3 × 10⁹ CFU/mL) and extracting with twice the volume of acetone, followed by concentration using a rotary evaporator to obtain the cell-free extract of the supernatant. Simultaneously, the bacterial cells were soaked in 1 mL of acetone for 24 h and subsequently ultrasonicated until the solution became clear, yielding the cell extract. The two extracts were then combined to obtain the YJ-2 active extract. Following this, 5 µL of the strain YJ-2 bacterial solution or 10 µL of the active extract was added at a distance of 25 mm on all four sides and cultured at 25 °C for 4–6 days. The diameter of the pathogenic fungi colony was measured using the cross method [43], and the diffusion area and inhibition rate of the pathogenic fungi were calculated. Each treatment was repeated three times, and the average value was taken. The inhibition percentage was calculated using the following formula:

Inhibition (%) = (r1 − r2)/r1 × 100,(1)

where r1 is the colony diameter of the pathogenic fungi in the control group, and r2 is the colony diameter of pathogenic fungi in the presence of endophytic bacteria.

### 2.8. Effect of Strain YJ-2 Active Extract on the Hyphal Morphology of V. mali

Using the plate confrontation culture method described above, *V. mali* was grown on a PDA plate for 4–6 days. A pathogen disk with a diameter of 5 mm was then transferred to the center of the PDA plate. Then, 10 µL of active extract was added at a distance of 25 mm and cultured at 25 °C for 4–6 days. The hyphae of *V. mali* at the edge of the inhibition zone formed by the antifungal effect of strain YJ-2 active extract were selected and observed under a microscope. The hyphae with normal growth on non-antifungal surfaces were used as the control group.

### 2.9. Stability Analysis of the Active Extract of Strain YJ-2

The stability of strain YJ-2 active extract was evaluated as previously described [44]. A PDA plate was inoculated with *V. mali* cakes (d = 5 mm) at the center of the plate, and then a sterile hole puncher (d = 5 mm) was used to punch holes 2.5 cm away from both sides of the cake. The active extract of strain YJ-2 was dissolved in 80% methanol and treated at 25 °C, 50 °C, 60 °C, and 100 °C for 30 min. The pH value of the active extract was adjusted to 2, 4, 6, 8, 10, and 12 using 1 mol/L HCl and 1 mol/L NaOH. Both 1 mol/L HCl and 1 mol/L NaOH were used as controls. Then, each sample (200 µL) was added to each hole and cultured at 25 °C for 4–6 days. All of the experiments were performed three times.

### 2.10. Experiment on Prevention of the Infection of V. mali in Apple Tree Branches

The scald inoculation method was used to determine the preventive effect of strain YJ-2 on the infection of *V. mali* in apple tree branches, as previously described [45]. Several perennial “Fuji” apple branches (d = 12–18 mm) with a length of 25 cm were collected. They were rinsed with water and disinfected for 10 min with 75% ethanol. Then, the branches were rinsed with sterile water three times and sealed with wax at both ends. The branches were then scalded with a burned iron nail cap (d = 5 mm), and the wounded area of the branches was exposed to a bacterial solution of strain YJ-2 or its various dilutions of its active extract stock solution (2, 5, 10, 20, 50, and 100 times dilution). The branches were then dried and inoculated with the cultured *V. mali* cakes (d = 5 mm) for 5 days. Each branch had an inoculation point. Five branches were treated with sterile water as a control for each group. After 7 days of dark culture with moisturizing at 25 °C, the length of the lesions was determined using the cross method, and the lesion area was calculated to evaluate the control effect of strain YJ-2.

S = (1/4π) × d1 × d2; P = [(S1 − S2)/S1] × 100%,(2)

where d1 is the lesion length diameter, d2 is the short diameter of the lesion, S1 is the colony area of the pathogenic fungi in the control group, and S2 is the colony area of the pathogenic fungi in the presence of endophytic bacteria.

### 2.11. Potted Anti-Efficiency Experiment of Strain YJ-2 Bone Gum Agent

The treatment effect of strain YJ-2 on potted apples after infection with *V. mali* was determined by referring to the scald inoculation method [45]. Here, we developed a bone colloid agent with strain YJ-2 as the main active ingredient (333.3 g bone gel, 3.33 g calcium carbonate, 333.3 mL water, 166.7 mL glycerol, and 500 mL strain YJ-2 fermentation broth) for subsequent treatment efficacy experiments. Five-year-old dwarf “Fuji” apple trees were planted, and the experiment was initiated after the trees sprouted new green leaves. Similarly, *V. mali* was cultured on a PDA plate for 5 days and then made into a cake (d = 5 mm) for later use. The branches of the apple saplings were burned with a burnt iron nail cap (d = 5 mm). The *V. mali* cake was then inoculated at the scald, and the *V. mali* cake was fixed on the branches with plastic wrap. Each sapling was set up with three inoculation points. The apple tree saplings were covered with transparent plastic bags and cultivated at room temperature under natural light to allow *V. mali* to infect the apple tree. During this period, water was sprayed on the apple tree every day to fully moisturize the sealed environment. After the wounds in the apple tree were inoculated for 5 days with *V. mali*, the infection was successful, which was obvious by the formation of typical rot disease lesions. Next, the infected area was scraped off the epidermis of the tree with a blade, and the infected apple trees were divided into three groups. The first group did not receive any treatment. The second group had the substrate applied to the infected area, and the third group had the strain YJ-2 bone gum agent applied to the infected area. After applying the strain YJ-2 bone gum agent, it was cultured under natural light and applied once every 10 days. The survival rate of the apple trees was observed and recorded to evaluate the treatment effect after 60 days.

### 2.12. Potted Anti-Efficiency Experiment of Strain YJ-2 Seed-Coating Agent

To obtain the *A. solani* fermentation broth, *A. solani* grown for 5 days was inoculated into PDA liquid medium and cultured at 25 °C with shaking at 200 rpm for 10–15 days until the mycelium bulb filled the medium. The prepared *A. solani* fermentation broth was added to sterile nutrient soil at a ratio of 500 mL/kg. We then prepared a seed-coating agent with strain YJ-2 as the main ingredient (200 mL of 4% chitosan solution, 640 mL of 4% polyvinyl alcohol solution, and 160 mL of strain YJ-2 fermentation broth), and 30 dwarf potted tomato seeds (Beijing Dongsheng Seed Industry Co., Ltd., Beijing, China) were added to 50 mL of the coating agent substrate or strain YJ-2 seed-coating agent. Once they were completely wrapped with seed-coating agent, they were dried for use. The seeds coated with strain YJ-2 seed-coating agent and substrate coating agent and the untreated control group were sown in nutrient soil with *A. solani* fermentation broth, as described above. Each treatment group had three biological replicates, and 10 seeds were sown in each replicate. The seed germination rate, plant root length, plant height, fresh weight, dry weight, and growth vigor of each treatment group were measured.

### 2.13. Potted Anti-Efficiency Experiment of Strain YJ-2 Wettable Powder

Tomato seedlings cultured for 50 days were used as experimental materials for the anti-efficiency experiment. The anti-efficiency experiment method was performed as previously described [46]. Briefly, to obtain the *A. solani* fermentation broth, *A. solani* grown for 5 days was inoculated into PDA liquid medium and cultured at 25 °C with shaking at 200 rpm for 10–15 days until the mycelium bulb filled the medium. The fermentation broth from a 15-day-old *A. solani* culture was collected and shaken with an oscillator for 2 min to shed the spores from the hyphae. After filtering the fermentation liquid of *A. solani* using sterile skim cotton, the supernatant was removed by centrifuging at 802× *g* for 5 min, and the precipitated spores were resuspended with 20% glycerol to obtain the *A. solani* spore solution. Wounds on tomato leaves were cut using a sterile blade, and then *A. solani* spore solution and 20 times the volume of strain YJ-2 wettable powder were sprayed on the scratched leaves for 3 days. According to the spraying order of *A. solani* spore solution and strain YJ-2 wettable powder, the experiment was divided into a preventive and therapeutic study of strain YJ-2 wettable powder on tomato early blight. The experiment was divided into eight treatment groups—A1: applied strain YJ-2 wettable powder and then inoculated *A. solani* spore solution; A2: inoculated *A. solani* spore solution and then applied strain YJ-2 wettable powder; A3: applied strain YJ-2 wettable powder; B1: applied wettable powder matrix and then inoculated *A. solani* spore solution; B2: inoculated *A. solani* and then applied wettable powder matrix spore solution; B3: applied wet powder matrix; CK1: inoculated *A. solani* spore solution; and CK2: untreated control group. The treated tomato seedlings were wrapped in plastic and incubated in a dark environment for 14 d. The incidence of tomato early blight, the incidence grade of tomato seedlings, the incidence area, and the prevention effect of strain YJ-2 wettable powder were measured. The incidence grade was scored according to the percentage of leaf lesion area in the whole leaf area [47]: Grade 0: none, Grade 1: less than 5%, Grade 3: 6–10%, Grade 5: 11–20%, Grade 7: 21–50%, and Grade 9: more than 50%. The incidence grade of 50 leaves was randomly investigated in each treatment. Disease index = 100 × Σ [(number of diseased leaves at all levels) × (representative value at all levels)]/[(total number of leaves surveyed) × (the highest representative value)].

### 2.14. Colonization of Strain YJ-2 in the Root and Shoot Parts of Tomato and Wheat Plants

The strain YJ-2 (pBBR-*gfp*-mut3) was constructed as previously described [48], with modifications. The pBBR-*gfp*-mut3 vector (GFP expression vector) was transferred to *E. coli* S17-1. *Escherichia coli* S17-1(pBBR-*gfp*-mut3) was used as the donor strain, and YJ-2 was used as the recipient strain. Both strains were cultured for 16 h, after which the donor and recipient strains were each inoculated into fresh tryptic soy broth (TSB) at 1% (*v*/*v*) and cultured until they reached the logarithmic growth phase. Subsequently, the donor and recipient strains were mixed at a defined ratio and incubated on the plate at 37 °C for 48 h. The strain YJ-2 (pBBR-*gfp*-mut3) colonies were selected after plating on TSB antibiotic plates containing both kanamycin (100 μg/mL) and gentamicin (100 μg/mL) and screening for GFP expression. The pBBR1MCS-5 (empty vector) was used as a control. The antifungal activity of strain YJ-2 (pBBR-*gfp*-mut3) was detected according to previous research methods [27].

The colonization test of strain YJ-2 (pBBR-*gfp*-mut3) in tomato seedlings (*Solanum lycopersicum* ‘Red Bonsai’, Beijing Dongsheng Seed Industry Co., Ltd., Beijing, China) was performed as previously reported [49]. Colonization experiments were conducted with dwarf tomato seedlings potted for 50 days. The soil was gently removed at the junction of the tomato rhizomes, and a sterile blade was used to create wounds on the roots and stems at the junction of the tomato rhizomes. The strain YJ-2 (pBBR-*gfp*-mut3) or strain YJ-2 (pBBR1MCS-5) bacterial solution was slowly poured along the tomato stems to the roots of the plant, and then the junction of rhizomes was covered with the soil. Each tomato was subjected to 100 mL of strain YJ-2 (pBBR-*gfp*-mut3) or strain YJ-2 (pBBR1MCS-5) bacterial solution and cultured at 25 °C for 21 days, and the control group was treated with the same volume of water. Tomato rhizomes were collected, the soil was rinsed with sterile water, and temporary sheets were made. The colonization of strain YJ-2 (pBBR-*gfp*-mut3) in the tomato roots and stems was observed under a fluorescence microscope. The same experimental procedure was used to detect strain YJ-2 (pBBR-*gfp*-mut 3) in wheat.

To further investigate the colonization ability of strain YJ-2 (pBBR-*gfp*-mut3), the colonization of strain YJ-2 (pBBR-*gfp*-mut3) in the tomato growth cycle was observed using the seed-coating agent method. Here, we prepared strain YJ-2 (pBBR-*gfp*-mut3) seed-coating agent, selected complete tomato seeds, put them in YJ-2 (pBBR-*gfp*-mut3) seed-coating agent, seeded them after complete wrapping and drying, and then cultured the seeds at 25 °C. The strain YJ-2 (pBBR1MCS-5) seed-coating agent and water treatment were used as controls. Tomato rhizomes were collected during germination, as well as the seedling, flowering, and fruiting stages. The soil was rinsed with sterile water, and temporary sheets were made. The colonization of strain YJ-2 (pBBR-*gfp*-mut3) in tomato seedlings at different growth stages was visualized using a blue light excitation fluorescence microscope (wavelength 420–485 nm).

### 2.15. Bioinformatics Analysis of Biocontrol

GeneMarkS (version 4.17) software was used to predict coding genes for the newly sequenced genome [50]. IslandPath-DIOMB software (version 0.2) was used to predict gene islands based on the sequences [51]. The general database was used to compare the protein sequences of predicted genes with the Perform Diamond comparison on each functional database. The comparison results were filtered, and the comparison result with the highest score for annotation was selected. Then, the Non-Redundant Protein Database (NR), Gene Ontology (GO), Kyoto Encyclopedia of Genes and Genomes (KEGG), and Cluster of Orthologous Groups of proteins (COG) databases were used to predict gene functions. AntiSMASH (version 4.0.2) was used to predict the genome secondary metabolite synthesis gene clusters by comparing with known biosynthetic gene clusters (http://antismash.secondarymetabolites.org/, accessed on 25 March 2025) [52].

### 2.16. Construction of Mutant and Complementary Strains

The knock-out plasmid was constructed based on methods from previously reported studies, with some modifications [53,54]. Briefly, using pDM4-*Δ6563* as an example, to construct the recombinant suicide plasmids for deletion, 1058 bp upstream and 1030 bp downstream of the *6563* gene were amplified using the primer pairs *6563* upF/*6563* upR and *6563* lowF/*6563* lowR, respectively (Supplementary Table S1). The upstream and downstream PCR fragments were ligated using overlapping PCR, and the resulting PCR products were inserted into the SacI/XbaI sites of the suicide vector pDM4 to yield the plasmid *p-6563*. Then, pDM4-*Δ6563* was transformed into *E. coli* S17-1 and conjugated with *B. stagnalis* YJ-2 to obtain single cross-over mutants. The *Δ6563* mutant strain was obtained through sucrose lethal screening and resistance screening. We used the same approach to obtain *Δ2107* and *Δ2117* mutant strains.

Using the construction of *Δ6563* complementary strain as an example, the YJ-2_GM006563 operon gene fragment was first amplified using primers *6563*F/*6563*R. The successfully amplified YJ-2_GM006563 operon gene fragment and the complementary vector pME6032 were simultaneously digested (EcoRI/Bgl II), ligated together, and then transformed into *E. coli* TG1. The positive clones were screened, and the recombinant complementary vector pME6032-*6563* was obtained. Subsequently, the recombinant vector was electroporated into the *Δ6563* mutant by electric shock transformation, and the genetically complementary strain was obtained by resistance screening. We used the same approach to obtain all complementary strains.

### 2.17. Identification of Genes Involved in the Antifungal Activity of Strain YJ-2

The antifungal effects of strain YJ-2, *Δ6563*, *Δ2107*, and *Δ2117* on *V. mali* were determined using the plate confrontation culture method. The mycelial cake of *V. mali* was inoculated face down in the center of a PDA plate. Then, 3 μL of strain YJ-2, *Δ6563*, *Δ2107*, or *Δ2117* bacterial solution was inoculated on the plate symmetrically at 2.5 cm on all four sides. The antifungal results were observed after 4–6 days of culture at 25 °C. The diameter of the *V. mali* colony was measured using the cross method [43], and the diffusion area and inhibition rate of *V. mali* were calculated. Each treatment was repeated three times, and the average value was taken. The inhibition percentage (I) was calculated using the following formula:

Inhibition (%) = (S1 − S2)/S1 × 100,(3)

where S1 is the colony area of pathogenic fungi in the control group, and S2 is the colony area of pathogenic fungi in the presence of endophytic bacteria.

### 2.18. Statistical Analysis

All experiments were performed in parallel at least three times and repeated independently twice. One-way ANOVA was performed by using IBM SPSS statistics 26.0 software (IBM Corp., Armonk, NY, USA) to compare multiple groups. The data are expressed as mean ± standard deviation, and the differences were considered statistically significant at *p* < 0.05. Adobe Illustrator 2020 software was used to draw graphs. The data between the two groups were compared using Dunnett’s new multiple range method in GraphPad Prism version 9.00 software (GraphPad Software, San Diego, CA, USA).

## 3. Results

### 3.1. Isolation of Strain YJ-2

A total of 25 bacterial strains were isolated from the collected soil, and only the *B. stagnalis* strain YJ-2 had the strongest inhibitory activity against *V. mali,* with an inhibition rate of 97.93% ± 0.54% (Supplementary Table S2). Therefore, this study focused on YJ-2. YJ-2 had been stored in the China Center for Type Culture Collection with the storage number CCTCC No: M20232448.

### 3.2. Identification of Strain YJ-2

**Figure 1:**
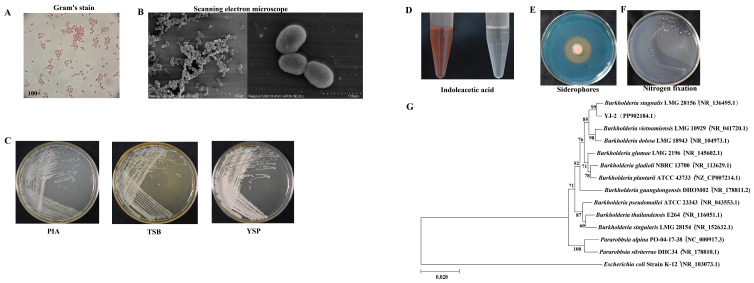
Morphological observation, biological characteristic detection, and construction of the genomic evolutionary tree of YJ-2. (**A**) Morphological characteristics of YJ-2 under a light microscope after Gram staining. (**B**) Morphological characteristics of YJ-2 under an electron microscope. (**C**) The colony morphology of YJ-2 on PIA medium, TSB medium, and YSP medium. (**D**) IAA production, (**E**) siderophore production, and (**F**) nitrogen fixation of YJ-2. (**G**) Phylogenetic tree of YJ-2 constructed based on 16S rRNA gene sequences.

YJ-2 is a Gram-negative bacterium (Figure 1A) and was observed under a scanning electron microscope to be short, rod-shaped, and without flagella (Figure 1B). The colony morphology of YJ-2 on PIA, TSB, and YSP medium appeared small, rough, opaque, and lacked flagella. The colonies had neat and concave edges and appeared as milky round colonies 1–2 mm in size (Figure 1C). This strain possessed the ability to generate IAA, with a yield of approximately 8.758 mg/L (Figure 1D and Supplementary Figure S1). A CAS plate was used to test the ability of YJ-2 to produce siderophores, and we observed an obvious orange halo produced on the CAS plate, indicating that YJ-2 can produce siderophores (Figure 1E). The nitrogen-fixing ability of YJ-2 was assessed using a nitrogen-free medium. The results demonstrated that YJ-2 is a strain with nitrogen-fixing ability (Figure 1F).

The 16S rRNA gene sequence of YJ-2 was sequenced by Beijing AugCT Biotechnology Co., Ltd., China, and the effective length of the 16S rRNA gene sequence was 1495 bp [(National Center for Biotechnology Information, NCBI) nucleic acid sequence number: PP902184.1]. The sequence similarity search and alignment in the NCBI database revealed that YJ-2 had a very high similarity to *Burkholderia* sp., indicating that YJ-2 is a member of *Burkholderia* sp. A phylogenetic tree was created using the maximum likelihood method of Bio-Edit and MEGA X64 software, showing that YJ-2 shared the same clade as *Burkholderia stagnalis* LMG 28156 on the phylogenetic tree, with the highest sequence identity of 99.72% (Figure 1G), indicating their close relationship. Therefore, strain YJ-2 was identified as *Burkholderia* sp. YJ-2 by combining the morphological and cultural characteristics.

### 3.3. Antifungal Spectrum of B. stagnalis YJ-2

**Figure 2:**
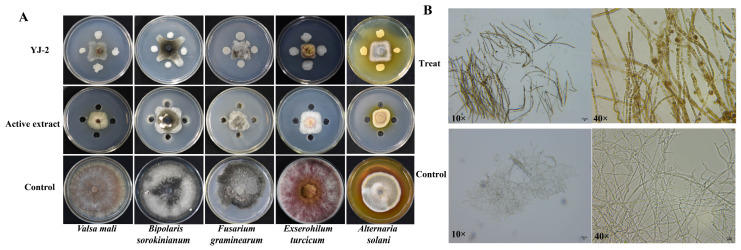
Antimicrobial effects of *Burkholderia* sp. YJ-2 and its active extract on the hyphal morphology of *V. mali*. (**A**) Inhibitory effect of *Burkholderia* sp. YJ-2 and its active extract on five plant pathogenic fungi. (**B**) Effects of the active extract of *Burkholderia* sp. YJ-2 on the hyphal morphology of *V. mali* during confrontation culture.

The antifungal activity of *Burkholderia* sp. YJ-2 and active extract of *B. stagnalis* YJ-2 on five pathogenic plant fungi (*V. mali*, *B. sorokinianum*, *E. turcicum*, *F. graminearum*, *A. solani*) was assessed using a plate confrontation method. It was found that *Burkholderia* sp. YJ-2 and active extract of *Burkholderia* sp. YJ-2 had obvious inhibitory effects on all five pathogenic plant fungi tested (Figure 2A). Among them, the maximum inhibitory rate of *Burkholderia* sp. YJ-2 on *F. graminearum* was 91.81% ± 0.16%, and the minimum inhibitory rate of *B. sorokinianum* was 82.80% ± 0.43%. The maximum inhibitory rate of the active extract of *Burkholderia* sp. YJ-2 on *V. mali* was 91.92% ± 0.05%, and the minimum inhibitory rate of *B. sorokinianum* was 82.46% ± 0.55% (Supplementary Table S3).

### 3.4. Hyphal Morphology Changes

Previous results demonstrated that *Burkholderia* sp. YJ-2 had a broad spectrum of antifungal activity against plant pathogenic fungi; however, it was unclear whether this antifungal effect directly affected the hyphae of plant pathogenic fungi. Therefore, in this study, *V. mali* was used as an indicator strain to explore the effect of the active extract of *Burkholderia* sp. YJ-2 on its hyphal morphology. The hyphae of *V. mali* at the edge of the inhibitory circle formed by the active extract of *Burkholderia* sp. YJ-2 were selected under the microscope, and the hyphae growing normally on the non-confrontation surface were used as the control. The results are shown in Figure 2B. When challenged by the active extract of *Burkholderia* sp. YJ-2, the hyphae showed fewer branches, fragmented cells, and darker colors. This suggested that the active extract of *Burkholderia* sp. YJ-2 altered the hyphal morphology of *V. mali.* Taken together, these results indicate that *Burkholderia* sp. YJ-2 has broad-spectrum antifungal activity against pathogenic plant fungi and inhibits the growth of pathogenic fungi by changing the structure of the hyphae.

### 3.5. Assessment of the Stability of the Active Extract of Burkholderia sp. YJ-2

The biological agents used in agriculture have many characteristics, among which stability is of great concern because good stability is one of the essential conditions for the promotion and use of biological agents. Therefore, this study investigated the stability of the active extract of *Burkholderia* sp. YJ-2. The results are shown in Table 1. After various temperature treatments, the activity of *Burkholderia* sp. YJ-2 active extract still existed. The antifungal activity of the active extract decreased significantly only when the temperature was above 90 °C and the pH ≥ 10. The above results indicated that the antifungal activity of the active substance of *Burkholderia* sp. YJ-2 is resistant to changes in temperature and acidity.

### 3.6. Evaluation of the Biocontrol Ability of Burkholderia sp. YJ-2 Against Pathogenic Plant Fungi

**Figure 3:**
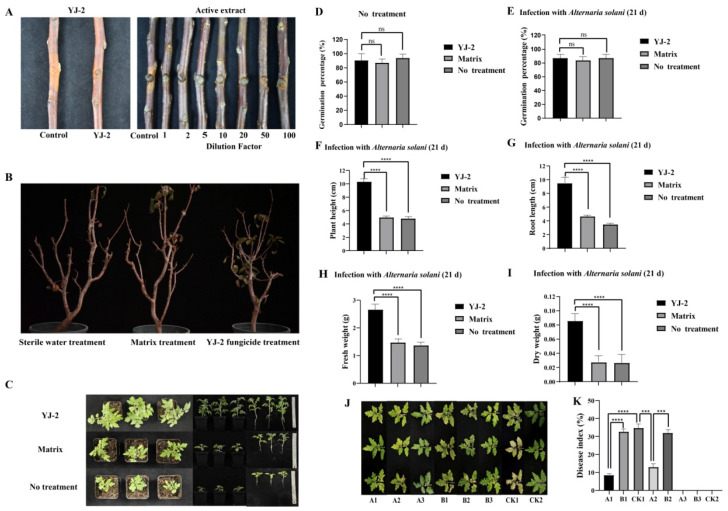
Biological control efficacy of *Burkholderia* sp. YJ-2 on pathogenic plant fungi. (**A**) Prevention of *V. mali* infection in collected apple tree branches. (**B**) Potted apple seedlings infected with *V. mali*. (**C**–**I**) Potted plant experiment assessing the control effect of *Burkholderia* sp. YJ-2 seed-coating agent. (**J**,**K**) Potted plant experiment assessing the control effect of *B. stagnalis* YJ-2 wettable powder. The error bars represent the standard deviations. Student’s *t* test; ***: *p* < 0.001; ****: *p* < 0.0001; ns, no significant difference; *n* = 5.

To study the effect of *Burkholderia* sp. YJ-2 against plant fungal diseases in vivo, we first analyzed the prevention effect of *Burkholderia* sp. YJ-2 in an apple tree isolated branch infection model of *V. mali*. The pre-application of *Burkholderia* sp. YJ-2 effectively prevented the infection of *V. mali* on the isolated apple tree branches, thus effectively inhibiting the expansion of *V. mali* spots on the isolated branches of apple trees (Figure 3A and Supplementary Table S4). The prevention effect reached 91.13% ± 0.38% (Figure 3A and Supplementary Table S4). Similarly, the pre-application of the active extract of *Burkholderia* sp. YJ-2 also effectively prevented *V. mali* infection, thus effectively inhibiting the expansion of *V. mali* spots on the isolated apple tree branches. The active extract, two times-diluted active extract, and five times-diluted active extract were 94.81% ± 0.18%, 94.38% ± 0.05%, and 93.16% ± 0.04% effective, respectively, in preventing *V. mali* infection in the isolated apple tree branches. Statistical analysis showed that neither of the diluted treatments differed significantly from the undiluted extract (*p* > 0.05). The prevention was above 80% when the dilution reached 20 times (Figure 3A and Supplementary Table S4), implying that the active extract by *Burkholderia* sp. YJ-2 has greater efficacy.

To verify the therapeutic effect of *Burkholderia* sp. YJ-2 on apple trees suffering from *V. mali* infection, a model of infection using potted apple seedlings was established. We used *V. mali* to infect healthy apple tree seedlings and smeared fungicide made from *Burkholderia* sp. YJ-2 onto the seedlings. After 30 days of treatment, the survival rate of these apple tree seedlings reached 83.33%, and the survival rate of apple tree seedlings in the base material treatment group and sterile water treatment group was only 33.33%, indicating that the fungicide made from *Burkholderia* sp. YJ-2 had a significant control effect on *V. mali* infection (Figure 3B and Supplementary Table S5).

Similarly, we used tomato plants to evaluate the control effect of *Burkholderia* sp. YJ-2 on early blight in tomatoes. Tomato seeds treated with *Burkholderia* sp. YJ-2 seed-coating agent, matrix-coating agent, or water treatment (negative control) were sown in soil mixed with *A. solani* fermentation broth. After 21 days, the growth of tomato seeds grown with the *Burkholderia* sp. YJ-2 seed-coating agent was significantly better than that of the matrix-coating agent group and water-treated group (Figure 3C). In the absence of inoculation with *A. solani* (Figure 3D), there was no significant difference in the seed germination rate between treatment groups, indicating that *Burkholderia* sp. YJ-2 seed-coating agent exhibited no negative effects on the germination of tomato seeds. After inoculation with *A. solani* (Figure 3E), although there was no significant difference in seed germination rate among all treatment groups, the plant height (Figure 3F), root length (Figure 3G), fresh weight (Figure 3H), and dry weight (Figure 3I) of the tomato seedlings in the *Burkholderia* sp. YJ-2 seed-coating group were significantly higher than those in the matrix-coating group and water treatment group. In conclusion, the results demonstrated that *Burkholderia* sp. YJ-2 seed-coating agent effectively protected tomato seedlings from *A. solani* infection.

We also used tomato plants to assess the control effect of *Burkholderia* sp. YJ-2-infused wettable powder on early blight in tomatoes. This experiment included eight treatment groups, which, according to the treatment order, assessed both the preventive effect of *Burkholderia* sp. YJ-2 wettable powder on tomato early blight (A1, B1, and CK1) and the therapeutic effect (A2, B2, and CK1). Compared with the matrix-treated and control groups, the disease spots of tomato seedlings treated with *Burkholderia* sp. YJ-2 wettable powder prior to *A. solani* infection were significantly reduced (Figure 3J,K). Overall, the disease index of tomato seedlings pre-treated with the *Burkholderia* sp. YJ-2 wettable powder was significantly reduced. Similarly, tomato plants treated with *Burkholderia* sp. YJ-2 wettable powder after *A. solani* infection (A2, B2, and CK1) also showed similar results (Figure 3J,K). Furthermore, the disease index of the A1 prophylactic treatment group was significantly lower than that of the A2 treatment group, indicating that *Burkholderia* sp. YJ-2 wettable powder had a better preventative effect on tomato early blight than a therapeutic effect. Compared with the A3, B3, and CK2 groups, the other groups infected with *A. solani* showed disease symptoms, indicating that the disease was caused by inoculated *A. solani* (Figure 3J,K). In conclusion, our results demonstrated that *Burkholderia* sp. YJ-2 wettable powder exhibits suitable preventive and therapeutic effects on tomato early blight.

### 3.7. Analysis of the Colonization Ability of Burkholderia sp. YJ-2 in Plants

The application value of *Burkholderia* sp. YJ-2 in the biological control of plant fungal diseases was determined. The colonization of *Burkholderia* sp. YJ-2 in tomato and wheat plants was assessed using GFP fluorescence labeling. The antifungal activity of *Burkholderia* sp. YJ-2 (pBBR-*gfp*-mut3) and *Burkholderia* sp. YJ-2 (pBBR1MCS-5) was compared using the plate confrontation method. We found no significant difference in the antifungal activity of *Burkholderia* sp. YJ-2 (pBBR-*gfp*-mut3) against pathogenic plant fungi, such as *F. graminearum*, *V. mali*, *B. sorokinianum*, and *A. solani*, as compared with *Burkholderia* sp. YJ-2 (pBBR1MCS-5). This result indicates that the addition of the GFP fluorescent-labeled plasmid, pBBR-*gfp*-mut3, did not affect the antifungal activity of *Burkholderia* sp. YJ-2 (Supplementary Figure S2). Therefore, we were able to use the *Burkholderia* sp. YJ-2 (pBBR-*gfp*-mut3) strain to analyze the colonization of *Burkholderia* sp. YJ-2 within the plant.

**Figure 4:**
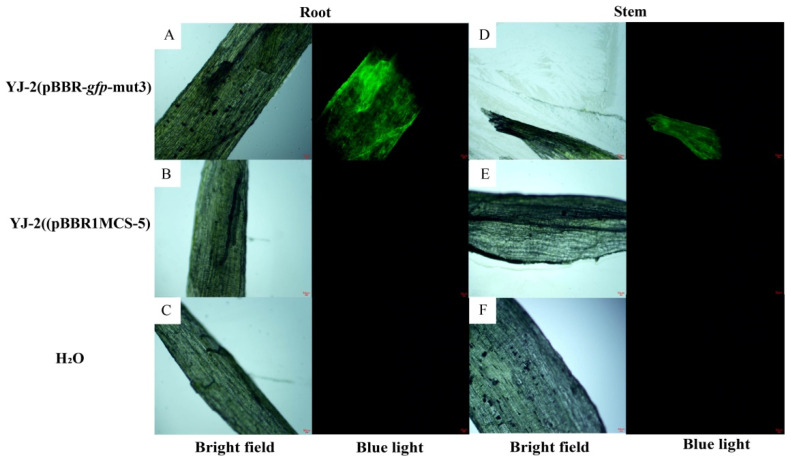
Colonization of *Burkholderia* sp. YJ-2 (pBBR-*gfp*-mut3) in tomato plants. *Burkholderia* sp. YJ-2 (pBBR-*gfp*-mut3)-treated tomato seedling root slices (**A**,**D**) were observed for fluorescence under white light and blue light using a fluorescence microscope. *Burkholderia* sp. YJ-2 (pBBR1MCS-5) (**B**,**E**) and water-treated (**C**,**F**) tomato seedling stem sections were observed for fluorescence under white light and blue light using a fluorescence microscope.

Tomato roots and stems were treated with *Burkholderia* sp. YJ-2 (pBBR-*gfp*-mut3) and subsequently harvested. Then, temporary sections were made on the plants, and the colonization of *Burkholderia* sp. YJ-2 (pBBR-*gfp*-mut3) was observed using a fluorescence microscope. As shown in Figure 4, after 21 days of treatment with *Burkholderia* sp. YJ-2 (pBBR-*gfp*-mut3), significant green fluorescence was observed in tomato root and stem sections (Figure 4A,D), while no significant green fluorescence was observed in tomato root and stem sections of the control group treated with *Burkholderia* sp. YJ-2 (pBBR1MCS-5) and H_2_O (Figure 4B,C,E,F). Similarly, wheat root and stem sections treated with *Burkholderia* sp. YJ-2 (pBBR-*gfp*-mut3) for 21 days also showed significant green fluorescence under a fluorescence microscope (Supplementary Figure S3A,D), while no significant green fluorescence was observed in wheat root and stem sections treated with *Burkholderia* sp. YJ-2 (pBBR1MCS-5) and H_2_O control groups (Supplementary Figure S3B,C,E,F).

In addition, we further investigated the colonization of *Burkholderia* sp. YJ-2 in the tomato during its growth cycle by growing tomato seeds coated with *Burkholderia* sp. YJ-2 (pBBR-*gfp*-mut3). We found that tomato seeds coated with *Burkholderia* sp. YJ-2 (pBBR-*gfp*-mut3) showed significant fluorescence in the roots and stems during the germination and seedling stages (Supplementary Figure S4), whereas minimal fluorescence was observed in the tomato roots and stems during the flowering and fruiting stages. *Burkholderia* sp. YJ-2 (pBBR1MCS-5) and H_2_O showed no obvious green fluorescence in tomato root and stem sections throughout the growth cycle (Figure S5). Taken together, these results indicate that *Burkholderia* sp. YJ-2 (pBBR-*gfp*-mut3) can stably colonize the roots and stems of tomato and wheat plants.

### 3.8. Bioinformatics Analysis of Burkholderia sp. YJ-2

#### 3.8.1. Genome Sequencing and Species Annotation of *Burkholderia* sp. YJ-2

**Figure 5:**
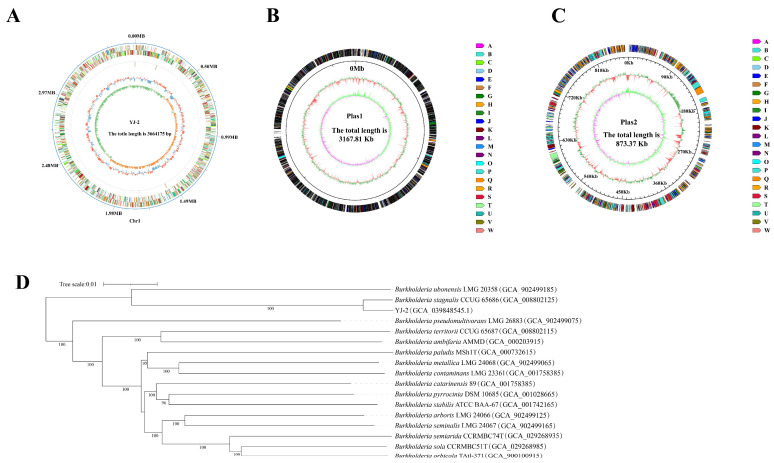
Circular genome map of *B. stagnalis* YJ-2. (**A**) Chromosome map, (**B**) plasmid loop 1, and (**C**) plasmid loop 2. (**D**) Phylogenetic tree of *B. stagnalis* YJ-2 based on the whole-genome sequence.

The genome of *Burkholderia* sp. YJ-2 was sequenced to characterize and identify its genomic features. The whole genome of *Burkholderia* sp. YJ-2 has been uploaded to the National Center for Biotechnology Information (NCBI) under accession number CP156685.1. The general genomic features of *Burkholderia* sp. YJ-2 were assessed and are summarized in Supplementary Table S6 and Figure 5A–C. The genome of *Burkholderia* sp. YJ-2 consisted of one chromosome and two plasmids (plasmids 1 and 2), with a total length of 7,705,355 bp, mean GC content of 67.69%, and 6858 coding genes. Additionally, 21 rRNA genes and 72 tRNA-coding genes were annotated in the chromosome sequence.

The NR database is a database for species classification. The amino acid sequences of all the proteins of *Burkholderia* sp. YJ-2 were aligned in the NR database using Diamond-2.1.8 software, and the gene annotations were performed according to the alignment results. The number of annotated species and genes was counted. The results demonstrated that *Burkholderia* sp. YJ-2 had the highest matching degree with *B. stagnalis*, and 6076 protein-coding genes in the genome of *Burkholderia* sp. YJ-2 were annotated into *B. stagnalis*. The TYGS online tool was used for genome alignment and similarity analysis, and a phylogenetic tree was constructed. Based on Genome BLAST Distance Phylogeny (GBDP), digital DNA-DNA hybridization (dDDH) values between *Burkholderia* sp. YJ-2 and different species were obtained (Figure 5D). The genetic relationship between *Burkholderia* sp. YJ-2 and *Burkholderia stagnalis* CCUG 65686 (accession number: GCA_008802125) was the closest, and its dDDH value reached 93.9% (Supplementary Table S7). From the genomic level, *Burkholderia* sp. YJ-2 belonged to *B. stagnalis*. The genomic dDDH value is one of the critical indicators for microbial species identification. When the dDDH is greater than 70, the two strains in comparison are identified to be the same species. Thus, *Burkholderia* sp. YJ-2 was named *Burkholderia stagnalis* YJ-2.

#### 3.8.2. Genome Analysis and Secondary Metabolite Synthesis Gene Cluster Prediction of *B. stagnalis* YJ-2

We previously found that *B. stagnalis* YJ-2 was an IAA-producing, nitrogen-fixing, and siderophore-secreting bacterium (Figure 1D–F). The genes in *B. stagnalis* YJ-2 associated with IAA, nitrogen fixation, and siderophore secretion were observed by functional annotation of the *B. stagnalis* YJ-2 genome (Supplementary Table S8). Specifically speaking, nine genes related to IAA were found in the *B. stagnalis* YJ-2 genome, comprising three amidase genes (YJ-2_GM000459, YJ-2_GM00070, and YJ-2_GM0030172), two monoamine oxidase genes (YJ-2_GM005295 and YJ-2_GM005308), and four aldehyde dehydrogenase genes (YJ-2_GM000027, YJ-2_GM003116, YJ-2_GM004030, and YJ-2_GM005704). Seven genes related to nitrogen fixation were identified in *B. stagnalis* YJ-2, comprising one glutamine synthetase (YJ-2_GM003448), two nitrogen regulatory protein-encoding genes (YJ-2_GM003575 and YJ-2_GM004035), two NADP-specific glutamate dehydrogenases (YJ-2_GM000921 and YJ-2_GM005966), and two nitrogen-fixing genes, *nifQ* and *nifU* (YJ-2_GM004349 and YJ-2_GM006795). We also found 17 genes related to siderophore synthesis in the genome of *B. stagnalis* YJ-2: one transcriptional regulator, *orbS* (YJ-2_GM004880); two biosynthetic non-ribosomal peptide synthetases, *orbI* and *orbJ* (YJ-2_GM004870 and YJ-2_GM004871); eight ornibactin-related synthesis genes, comprising the oxygenase *pvdA* and *obrG* (YJ-2_GM004868 and YJ-2_GM004878), the ornibactin biosynthetic protein-encoding genes, *obrL* and *obrK* (YJ-2_GM004865 and YJ-2_GM004869), siderophore synthase *obrF* (YJ-2_GM004866), ferric reductase protein-encoding gene *fhuF* (YJ-2_GM004875), and siderophore synthesis accessory protein-encoding gene *orbH* (YJ-2_GM004879 and YJ-2_GM006205); and six ornibactin transport-related genes, comprising iron complex outer membrane receptor protein *obrA* (YJ-2_GM004779 and YJ-2_GM004867) and ATP-binding transporter *orbBCDE* (YJ-2_GM004872, YJ-2_GM004874, YJ-2_GM004876, and YJ-2_GM004877). Thus, the annotation of these genes confirmed at the genetic level that *B. stagnalis* YJ-2 had the ability to produce IAA, siderophores, and fix nitrogen, suggesting that *B. stagnalis* YJ-2 had good potential for growth-promoting function.

We further analyzed the secondary metabolite synthesis gene clusters of *B. stagnalis* YJ-2 to determine if any might be involved in regulating the antifungal activities of YJ-2. We identified three biosynthetic gene clusters of known secondary metabolites, namely ornibactin, icosalide, and sinapigladioside. One of the twenty predicted secondary metabolite synthesis gene clusters of YJ-2 was related to the synthesis of the siderophore ornibactin and included the core biosynthetic genes YJ-2_GM002107, YJ-2_GM002109, and YJ-2_GM002117. The ornibactin family is a siderophore secreted by bacteria, such as *Burkholderia* [55]. In addition to promoting growth, siderophores also have the effect of antagonizing pathogenic fungi. There were two other predicted secondary metabolic gene clusters with high similarity (as high as 100%) to known gene clusters. One gene cluster was the non-ribosomal polypeptide (NRPS) icosalide. Icosalide is an alicyclic peptide antibiotic with antibacterial and cytotoxic effects [56], and its core biosynthetic gene in the *B. stagnalis* YJ-2 genome is YJ-2_GM002026. The other gene cluster was the other unspecified ribosomal synthesis, and the post-translational modification peptide product (RiPP) was sinapigladioside. Sinapigladioside is a natural product containing isothiocyanate, which exhibits antifungal and other biological activities and may have potential application value in agriculture and medicine. Its core biosynthetic genes in the *B. stagnalis* YJ-2 genome are YJ-2_GM006551 and YJ-2_GM006563. The remaining 17 gene clusters had low similarity to known gene clusters, which include a large number of unknown gene clusters and, as such, deserve further investigation (Table 2).

### 3.9. Identification of Genes Involved in the Antifungal Effects of YJ-2

**Figure 6:**
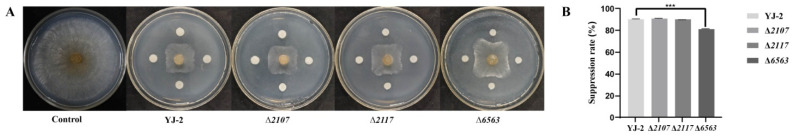
Antifungal effects of *B. stagnalis* mutants on *V. mali*. (**A**) Antifungal effects of YJ-2, *Δ2107*, *Δ2117*, and *Δ6563* on *V. mali*. (**B**) Inhibition rates of YJ-2, *Δ2107*, *Δ2117*, and *Δ6563* against *V. mali*. The error bars represent the standard deviations. Student’s *t* test; ***: *p* < 0.001; *n* = 3.

We previously predicted that the three secondary metabolic gene clusters were associated with antifungal activity, as they were homologous to the biosynthetic gene clusters of known secondary metabolites, such as icosalide, ornibactin, and sinapigladioside. Thus, we constructed knock-out mutants (*Δ2107*, *Δ2117*, and *Δ6563)* to determine the role these genes (YJ-2_GM002107, YJ-2_GM002117, and YJ-2_GM006563, respectively) play in the antifungal activity of *B. stagnalis*. We found that both *Δ2107* and *Δ2117* exhibited antagonistic activities against *V. mali* comparable to *B. stagnalis* YJ-2 (Figure 6). Interestingly, the antifungal effect of *B. stagnalis Δ6563* was 8.79% lower than that of *B. stagnalis* YJ-2, indicating that YJ-2_GM006563 is involved in the antifungal effect of *B. stagnalis* YJ-2. However, because the antifungal effect of *Δ6563* was only slightly reduced, there are obviously other genes also involved in this process (Figure 6 and Supplementary Figure S5).

## 4. Discussion

In this study, we isolated *B. stagnalis* YJ-2 with broad-spectrum anti-phytopathogenic fungal activity. Its bioactive extracts remained stable across a wide range of temperatures and pH levels. In vitro assays demonstrated that its metabolites disrupted the hyphal morphology of *V. mali*, leading to swelling, reduced branching, and increased pigmentation. This strain also produced IAA and siderophores and possessed nitrogen fixation ability. GFP fluorescence-based colonization analysis revealed that *B. stagnalis* YJ-2 stably colonized both tomato and wheat seedlings. A seed-coating agent formulated with *B. stagnalis* YJ-2 effectively protected tomato seedlings against early blight without affecting seedling germination. The *B. stagnalis* YJ-2 wettable powder demonstrated both preventive and therapeutic effects against tomato early blight, with preventive treatment displaying a more pronounced effect than therapeutic treatment. The bone glue-based *B. stagnalis* YJ-2 also exhibited a moderate control effect against apple Valsa canker. These results indicate that *B. stagnalis* YJ-2 possesses strong environmental adaptability and excellent biocontrol potential. The genome of B. stagnalis YJ-2 was assessed to further explore the biocontrol mechanism of *B. stagnalis* YJ-2. The genomic analysis revealed that it belongs to *B. stagnalis*. Twenty secondary metabolite gene clusters were predicted, including three with homology to known biosynthetic clusters, such as those involved in antifungal compound production (e.g., glucosinolate). Notably, the deletion of the YJ-2_GM006563 gene reduced the antifungal activity of *B. stagnalis* YJ-2 by 8.79%, suggesting a partial but non-exclusive role of the gene cluster (YJ-2_GM006534-YJ-2_GM006569) in inhibiting phytopathogenic fungi. These findings provide a foundation for the industrial application of *B. stagnalis* YJ-2, as well as future studies on its mechanisms against plant pathogens.

Biocontrol *Burkholderia* sp. could inhibit pathogenic plant fungi in multiple ways after colonizing and contributing to the growth of plants to varying degrees. For example, Sun et al. reported that *Burkholderia* sp. YZU-s230, with broad-spectrum antagonistic properties, promoted the growth of watermelon by increasing the main vine length, root length, and fresh weight [57]. Han et al. found that *Burkholderia pyrrocinia* A12 significantly enhanced the chlorophyll content, photosynthetic rate, transpiration rate, and root growth of *Nicotiana tabacum* [58]. In this study, we found that there are two IAA synthesis pathways in the genome of *B. stagnalis* YJ-2, which contain several genes (YJ-2_GM003017, YJ-2_GM003116, YJ-2_GM004030, YJ-2_GM005295, YJ-2_GM005308, YJ-2_GM005704, YJ-2_GM000027, YJ-2_GM000459, and YJ-2_GM000702). For the indole-3-acetamide (IAM) pathway, tryptophan monooxygenase catalyzes L-tryptophan to form IAM, and IAM forms IAA through the action of amidase. For the tryptamine (TPM) pathway, tryptophan decarboxylase catalyzes tryptophan to form TPM, and amine oxidase catalyzes TPM to produce indole-3-acetaldehyde (IAAld). IAAld is then catalyzed by acetaldehyde dehydrogenase to eventually form IAA. As the genome of *B. stagnalis* YJ-2 lacks genes encoding tryptophan monooxygenase and tryptophan decarboxylase, it is inferred that both IAM and TPM pathways are incomplete in this rhizosphere-associated strain. Notably, previous experimental assays confirmed that *B. stagnalis* YJ-2 can synthesize IAA, and tomato seedlings treated with *B. stagnalis* YJ-2 seed-coating agent grew significantly better than those in the matrix-coated and untreated groups, indicating a potential plant growth-promoting effect. However, due to the absence of uninfected control treatments, this effect cannot be conclusively attributed to growth promotion. Therefore, the potential growth-promoting effect of *B. stagnalis* YJ-2 on plants requires confirmation through future studies. *B. stagnalis* YJ-2 also demonstrates nitrogen-fixing ability on Ashby’s medium, which is consistent with most *Burkholderia* sp. strains. The YJ-2 genome contains seven nitrogen fixation-related genes (YJ-2_GM003448, YJ-2_GM003575, YJ-2_GM004035, YJ-2_GM004349, YJ-2_GM005966, YJ-2_GM000921, and YJ-2_GM006795). These findings not only reaffirm the nitrogen-fixing ability of *B. stagnalis* YJ-2 but also suggest its potential as a biological nitrogen-fixing chassis.

*Burkholderia* sp. exhibits a remarkable ability to produce secondary metabolites, most of which possess antimicrobial properties, including pyrrolnitrin [59], occidiofungin [60], and enacyloxin IIa [61]. There are three gene clusters—icosalide (YJ-2_GM002015-YJ-2_GM002048), ornibactin (YJ-2_GM002090-YJ-2_GM002133), and sinapigladioside (YJ-2_GM006534-YJ-2_GM006569)—identified in the *B. stagnalis* YJ-2 genome, which may also contribute to its antifungal secondary metabolite production. Icosalide is a rare, two-tailed alicyclic peptide antibiotic [56]. This compound was also reported to be synthesized by *Burkholderia gladioli* BCC0238 [62]. Recently, icosalide was detected in active extracts of both *Bacillus* and *Serratia* with antagonistic activity against *Aspergillus flavus*, and it may function by disrupting fungal cell membranes to defend against competitors [63]. Ornibactin is a tetrapeptide hydroxamic acid siderophore consisting of l-ornithine-d-hydroxy-aspartate-l-serine-l-or-nithine as the basic structural skeleton [64]. The ornibactin gene cluster of *B. stagnalis* YJ-2 shares 100% sequence identity with that of *B. stagnalis* MSMB735WGS [65], and its encoded proteins are highly homologous to those from *B. pyrrocinia* LWK2 [66]. We also detected ornibactin in this study, as evidenced by *B. stagnalis* YJ-2 producing a yellow halo on CAS medium. Aside from promoting plant growth, siderophores also have antimicrobial activity. Ornibactin is one of the primary types of siderophores produced by *Burkholderia* [67]. Increasing evidence suggests that the antimicrobial activity of these siderophores is independent and may also play a role in antagonizing bacterial or fungal pathogens [68,69]. Yu et al. [70] reported that although pure ornibactin could not be isolated and its fungistatic activity remains unclear, it was still identified as a key factor influencing the antifungal activity of *Burkholderia*. Sinapigladioside is a rare isothiocyanate-bearing natural product and a potent antifungal metabolite produced by the beetle symbiont *Burkholderia gladioli* HKI0739 [71]. This substance exhibits distinct ecological activities in different environments [71]. It can protect insect eggs from pathogens, but it also causes mushroom soft rot [72,73]. In this study, the extracted substances with antifungal activity remained stable under both acid and alkali conditions, consistent with the known acid resistance of lipopeptides [74,75], suggesting that *B. stagnalis* YJ-2 may produce lipopeptide-containing antimicrobial compounds. Notably, the core gene sequence in the icosalide gene cluster is nearly 15,000 bp, making it difficult to delete. As a result, we turned to validating core genes in the other two gene clusters. Although we successfully deleted two core genes, *Δ2107* and *Δ2117,* associated with the ornibactin gene cluster, deletion of these genes did not affect the ability of *B. stagnalis* YJ-2 to inhibit *V. mali*. The deletion of the biosynthetic gene YJ-2_GM006563, which is part of a sinapigladioside synthesis gene cluster in *B. stagnalis* YJ-2, led to a slight reduction in antifungal activity, indicating that the lipopeptides synthesized by these genes contributed partially, but not predominantly, to the antifungal properties of *B. stagnalis* YJ-2. Future research will be aimed at identifying additional key genes to uncover the antagonistic mechanism of *B. stagnalis* YJ-2 and to determine whether other pathways contribute to its antifungal effects.

In response to the crisis of agricultural losses caused by plant fungal diseases, it is imperative to develop biocontrol agents that are low-cost, practically applicable, and highly adaptable to diverse environmental conditions. Currently, Marrone Bioinnovation has developed a biopesticide formulation using inactivated *B. rinojensis* A396 as the active ingredient [76]. The commercial application of live *Burkholderia*-based biocontrol agents, however, is relatively limited. This study demonstrated that *B. stagnalis* YJ-2 can stably colonize plant roots and stems over extended periods, and it requires low-cost and simple culture conditions, has a short fermentation cycle, and produces antifungal active substances that are highly stable at different temperatures and pH levels. Thus, this study suggests that *B. stagnalis* YJ-2 is a promising candidate for development as a commercial biopesticide. It should be noted that the key value of a biological control agent lies in whether it can colonize plants over an extended time and continue to play a role in prevention and control. In this study, we simulated the orchard environment and formulated a bone-glue agent with *B. stagnalis* YJ-2 as the primary active ingredient to assist its better colonization in apple trees. To meet different application conditions and to further evaluate the practical application potential, formulations, such as wettable powders and seed coatings, have been developed. We confirmed that *B. stagnalis* YJ-2 can stably colonize the roots and stems of both tomato and wheat plants. It is worth noting that because of complex field conditions, such as temperature, humidity, and soil texture, biocontrol efficacy in laboratory settings may differ from real-world outcomes [77]. Environmental interference makes it difficult to maintain consistent product quality and efficacy, thereby posing significant challenges to commercial promotion. Given that *Burkholderia stagnalis* belongs to the *Burkholderia cepacia* complex, which includes opportunistic pathogens, biosafety considerations are critical for its application as a biocontrol agent. Future studies should focus on detailed risk assessments and ensure regulatory compliance to facilitate the responsible deployment of *B. stagnalis* YJ-2. In conclusion, *B. stagnalis* YJ-2 demonstrated strong potential as a novel, eco-friendly, and efficient biological control agent. To enhance its practical application in agriculture, further research should elucidate its antifungal mechanisms, conduct large-scale field trials, and evaluate its efficacy and ecological safety under long-term and diverse environmental conditions.

## 5. Conclusions

*B*. *stagnalis* YJ-2, a rhizobacterium isolated from *W. ilvensis*, exhibits potent broad-spectrum antifungal activity against key phytopathogens (e.g., *V*. *mali*). Its thermostable and pH-tolerant bioactive extracts disrupt hyphal integrity by inducing swelling, branching reduction, and pigmentation accumulation. Fluorescence labeling confirmed stable colonization in both tomato and wheat tissues. Formulations derived from YJ-2 demonstrated significant efficacy. Seed coatings protected tomato seedlings from *A. solani* without compromising germination, while wettable powders preferentially suppressed early blight via preventive action, and bone glue agent effectively inhibited the spread of apple tree rot on apple tree trunks. Genomic analysis revealed a 7,705,355 bp genome harboring 20 secondary metabolite clusters, including three homologous to known antifungal biosynthetic clusters (e.g., sinapigladioside, ornibactin, and icosalide). Crucially, the deletion of the YJ-2_GM006563 gene reduced the antifungal activity of ***B*. *stagnalis* YJ-2**, implicating partial but non-exclusive roles of this cluster in pathogenic plant fungi suppression. Hence, *B. stagnalis* YJ-2 has immense potential for biological control, which is of great significance for not only developing biological control agents against plant fungal diseases but also for promoting sustainable agricultural disease management.

## Figures and Tables

**Table 1:** Stability of the active extract of *Burkholderia* sp. YJ-2 at different temperatures and pH levels.

T/°C	Antimicrobial Activity	pH	Antimicrobial Activity
25	+++	2	+++
50	+++	4	+++
60	+++	6	+++
70	+++	8	+++
80	+++	10	+++
90	++	12	++
100	+	CK1	-
ND	ND	CK2	-

+++: strong antimicrobial activity; ++: moderate antimicrobial activity; +: weak antibacterial activity and -: no antimicrobial activity; ND: no data; CK1: 1 mol/L HCl; CK2: 1 mol/L NaOH.

**Table 2:** Secondary metabolite gene clusters of *B. stagnalis* YJ-2.

Clusters	Type	Gene ID	Similar Known Cluster	Similarity (%)
Cluster 1	Arylpolyene	YJ-2_GM000898-YJ-2_GM000935	APE-Vf	10
Cluster 2	Terpene	YJ-2_GM001765-YJ-2_GM001781	-	-
Cluster 3	RiPP-like	YJ-2_GM002008-YJ-2_GM002012	-	-
Cluster 4	NRPS	YJ-2_GM002015-YJ-2_GM002048	Icosalide A/icosalide B	100
Cluster 5	NRPS	YJ-2_GM002090-YJ-2_GM002133	Ornibactin C8/ornibactin C4/ornibactin C6	100
Cluster 6	NRPS	YJ-2_GM002209-YJ-2_GM002245	Aminochelin/azotochelin/protochelin	62
Cluster 7	Arylpolyene	YJ-2_GM002427-YJ-2_GM002464	APE-Ec	36
Cluster 8	Hserlactone, terpene	YJ-2_GM003941-YJ-2_GM003963	-	-
Cluster 9	NRPS-like, T1PKS, betalactone	YJ-2_GM004190-YJ-2_GM004214	-	-
Cluster 10	Terpene	YJ-2_GM004564-YJ-2_GM004582	N-acyloxyacylglutamine	50
Cluster 11	Phosphonate	YJ-2_GM004702-YJ-2_GM004730	Pf-5 pyoverdine	2
Cluster 12	HR-T2PKS	YJ-2_GM006205-YJ-2_GM006244	S56-p1	7
Cluster 13	RRE-containing, RiPP-like	YJ-2_GM005923-YJ-2_GM005932	-	-
Cluster 14	Ectoine	YJ-2_GM000056-YJ-2_GM000064	Kosinostatin	4
Cluster 15	NRPS, NRPS-like	YJ-2_GM006155-YJ-2_GM006226	Bolagladin A/bolagladin B	19
Cluster 16	Hydrogen-cyanide	YJ-2_GM006725-YJ-2_GM006735	-	-
Cluster 17	Hserlactone	YJ-2_GM006304YJ-2_GM006324	Bactobolin	23
Cluster 18	T1PKS	YJ-2_GM006380-YJ-2_GM006411	Difficidin	13
Cluster 19	Betalactone	YJ-2_GM006478-YJ-2_GM006504	Pacifibactin	10
Cluster 20	RiPP-like	YJ-2_GM006534-YJ-2_GM006569	Sinapigladioside	100

## Data Availability

The original contributions presented in this study are included in the article and Supplementary Materials. Further inquiries can be directed to the corresponding authors.

## Supplementary Materials

The following supporting information can be downloaded at: https://www.mdpi.com/article/10.3390/microorganisms13061289/s1, Figure S1: IAA standard curve; Figure S2: The antagonistic effects of *B. stagnalis* YJ-2 (pBBR-*gfp*-mut3) and *B. stagnalis* YJ-2 (pBBR1MCS-5) on plant pathogenic fungi; Figure S3: Colonization of B. stagnalis YJ-2 (pBBR-*gfp*-mut3) in wheat; Figure S4: Colonization of Burkholderia sp. YJ-2 (pBBR-*gfp*-mut 3) during the tomato growth cycle; Figure S5: Effect of loss of YJ-2-GM006563 in *B. stagnalis* YJ-2 on antagonism of plant pathogenic fungi; Table S1: List of primiers used in this study; Table S2: The inhibitory rates of the strain YJ-2 against *V. mali*; Table S3: Inhibitory effect of the strain YJ-2 on the growth of five plant pathogenic fungi on PDA plates; Table S4: The preventive effect of active extract from *Burkholderia* sp. YJ-2 on apple valsa canker infect in vitro branches; Table S5: *Burkholderia* sp. YJ-2 on treatment of potted apple tree infection with *V.mali*; Table S6: Genome features of *Burkholderia* sp. YJ-2; Table S7: Genomic characteristics of *Burkholderia* sp. YJ-2 phylogenetic tree members; Table S8: IAA, siderophore, and nitrogen fixation related genes of *B. stagnalis* YJ-2 [78,79,80,81,82,83,84,85,86,87,88].
