# Supplementary material for: Isolation and Identification of Burkholderia stagnalis YJ-2 from the Rhizosphere Soil of Woodsia ilvensis to Explore Its Potential as a Biocontrol Agent Against Plant Fungal Diseases
Source: Microorganisms. 2025 May 31;13(6):1289. doi: 10.3390/microorganisms13061289 (PMC12195442; doi:10.3390/microorganisms13061289)
Supplement: Supplementary file 1 [file microorganisms-13-01289-s001.zip › microorganisms-3645075-supplementary.pdf]

Isolation and Identification of *Burkholderia stagnalis* YJ-2 from the Rhizosphere Soil of *Woodsia ilvensis* to Explore Its Potential as a Biocontrol Agent Against Plant Fungal Diseases

Xufei Zhu [1,†], Wanqing Ning [1,2,†], Wei Xiao [1], Zhaoren Wang [1], Shengli Li [1], Jinlong Zhang [1], Min Ren [1], Chengnan Xu[1], Bo Liu[1], Yanfeng Wang[1], Juanli Cheng [1,2,*] and Jinshui Lin[1,2,*]

1 Shaanxi Key Laboratory of Research and Utilization of Resource Plants on the Loess Plateau, College of Life Sciences,Yan'an University, Yan'an, Shaanxi, China; 17806813772@163.com (X.Z.); 18387120837@163.com (W.N.); 18792388186@163.com (W.X.); zhaorenwang00@163.com (Z.W.); 18181017935@163.com (S.L.); zhangjinlong04@yau.edu.cn (J.Z.); minren_edu@163.com (M.R.); xuchengnan1981@sina.com (C.X.); liubo4552@126.com (B.L.); wyfcollege@sohu.com (Y.W.).
2 State Key Laboratory for Crop Stress Resistance and High-efficiency Production, Northwest A&F University,Yangling, Shaanxi, China
* Correspondence: chengjl@yau.edu.cn (J.C.), linjinshui@yau.edu.cn (J.L.)
† These authors contributed equally to this work.

Contents:

Supplementary Table S1

Supplementary Table S2

Supplementary Table S3

Supplementary Table S4

Supplementary Table S5

Supplementary Table S6

Supplementary Table S7

Supplementary Table S8

Supplementary Figure S1

Supplementary Figure S2

Supplementary Figure S3

Supplementary Figure S4

Supplementary Figure S5

Supplemental Materials

Table S1. List of primiers used in this study.

Primer	5′→3′ Sequences
Δ6563 upF	CTAGGAGCTCGGCTGCTCGAGCTGCTGATC
Δ6563 upR	GCAGCAGCTGGCGTCACGAGCATCTTGG
Δ6563 lowF	GACGCCAGCTGCTGCAGGCCGTCGAAGC
Δ6563 lowR	CTAGTCTAGACTCGAAGCCGTTGCGGATC
Δ2117 upF	CTAGGAGCTCGTGGCATCCGGCTTCCAG
Δ2117 upR	GTTCTTCGGGAACGGTTTGATCAACATC
Δ2117 lowF	CAAACCGTTCCCGAAGAACCGGATGGC
Δ2117 lowR	CTAGTCTAGAGCCACCTTCATACGCTGC
Δ2107 upF	CTAGGAGCTCGTTTTCCCATCTTTCGTG
Δ2107 upR	CATCAGCGAGAAATCCTTGAACTGCAGG
Δ2107 lowF	CAGTTCAAGGATTTCTCGCTGATGGAGT
Δ2107 lowR	CTAGTCTAGACTTCAAGAACCCGGTGATC
6563F	CTAGGAATTCATGGAACCCTGGCTTGGCGATC
6563R	CTAGAGATCTTCAGAAAGTAGGGGAAGC

*Underlined sites indicate restriction enzyme cutting sites added for cloning.

Table S2. The inhibitory rates of the strain YJ-2 against *V. mali*.

Strain	Lesion area/mm^2	Disease prevention effect/%
LI-1	1851±20	-
LI-2	1462±16	20.93±0.87
LI-3	1760±16	4.81±0.87
LI-4	1375±8.5	25.64±0.50
Shil-1	1609±9	12.98±0.49
Shil-2	1215±65	34.29±3.52
Shil-3	1298±6.5	29.80±0.35
XL-1	1848±8	-
XL-2	1443±22.5	21.96±0.22
XL-3	1600±8.5	13.47±0.50
XL-4	1794±51	2.97±2.76
XL-5	1842±7	-
XL-6	1603±23	13.30±1.24
XL-7	1303±19.5	29.53±1.05
XL-8	1655±2.5	10.49±0.14
YJ-1	1359±17	26.50±0.92
YJ-2	38±4.5	97.93±0.54
YJ-3	1375±5.5	25.64±0.30
SL-1	1755±11	5.08±0.59
SL-2	1412±26	23.63±1.41
SL-3	1605±12.5	13.20±0.68
ZJC-1	900±15.5	51.33±0.84
ZJC-2	1242±4.5	32.83±0.24
ZJC-3	1302±20	29.58±1.08
YML-1	1851±13.5	-
CK	1848±8.5	-

Table S3. Inhibitory effect of the strain YJ-2 on the growth of five plant pathogenic fungi on PDA plates.

Type	YJ-2		extract		CK
	Lesion area/mm2	Disease prevention effect/%	Lesion area/mm2	Disease prevention effect/%	
Valsa mali	559.67±10.84	97.77±0.03	498.33±10.84	98.02±0.05	25152.45±229.46
Bipolaris sorokinianum	1075.33±31.68	95.74±0.11	1100±55.71	95.64±0.18	25246.65±264.96
Exserohilum turcicum	626±54.63	96.42±0.36	575±18.78	96.71±0.11	17505.5±219.81
Fusarium graminearum	385.33±48.73	97.97±0.17	536±11.31	97.87±0.05	25152.45±229.46
Alternaria solani	530±56.36	95.96±0.38	354±8.49	97.29±0.04	13063.45±165.37

Table S4. The preventive effect of active extract from *Burkholderia* sp. YJ-2 on apple valsa canker infect in vitro branches.

Type	Diluted times	Average lesion lengths /mm	Average lesion widths/mm	Lesion area/mm^2	Disease prevention effect /%
Cell	-	4.5±0.54b	5.5±0.74b	24.75±0.40b	91.13±0.38
CK1	-	20.2±1.30a	13.8±0.67a	278.76±0.87a	
Active	1	8.55±0.15f	6.53±0.14f	55.82±1.93d	94.81±0.18a
extract	2	8.82±0.02f	6.85±0.05ef	60.45±0.55d	94.38±0.05a

	5	9.73±0.07f	7.58±0.10e	73.71±0.58d	93.16±0.04a
	10	13.38±0.77e	8.47±0.04d	113.40±7.04d	89.46±0.66b
	20	16.76±0.19d	8.87±0.09d	148.69±2.51cd	86.18±0.23c
	50	21.72±0.45c	11.35±0.12c	246.65±7.82c	77.07±0.73d
	100	29.46±0.63b	13.94±0.06b	410.60±7.77b	61.83±0.72e
CK2		36.33±2.66a	29.56±0.84a	1075.79±103.15a	-

Data with the different lowercase letters in the same column indicate significant difference at $P<0.05$ level.

Table S5. *Burkholderia* sp. YJ-2 on treatment of potted apple tree infection with *V.mali*.

Processing method	Infected trees	Dead trees	Surviving trees	survival rate/%
Control	18	12	6	33.33
Basic material	18	12	6	33.33
Ointment agents	18	3	15	83.33

Table S6. Genome features of *Burkholderia* sp. YJ-2.

category		Value
Genome size (bp)	chromosome	3,664,175
	Plasmid 1	3,167,812
	Plasmid 2	873,368
Sequence GC (%)	chromosome	67.79
	Plasmid 1	67.36
	Plasmid 2	67.94
tRNAs		72
5s rRNAs		7
16s rRNA		7
23s rRNA		7
sRNA		0
Genomics islands		19
Prophage		1
CRISPRs		3

Table S7. Genomic characteristics of *Burkholderia* sp. YJ-2 phylogenetic tree members.

Strain	Authority	Synonyms	Percent G+C	No. proteins	Bioproject accession	Biosample accession	Assembly accession ID	dDDH	Diff. G+C Percent
B. pseudomultivorans LMG 26883	Peeters et al. 2014[2]	*Burkholderia pseudomultivorans*	67.0	6712	PRJEB33447	SAMEA5795710	GCA_902499075	37.8	0.63
B. arboris LMG24066	Vanlaere et al.2008[3]	*Burkholderia arboris*	66.8	7364	PRJEB33447	SAMEA5780076	GCA_902499125	40.1	0.78
B. *catarinensis* 89	Bach et al. 2019[4]	*Burkholderia catarinensis*	66.4	6128	PRJNA338131	SAMN05521522	GCA_001883705	39.7	1.23
B. paludis MSh1T	Ong et al. 2016[5]	*Burkholderia paludis*	67.1	6963	PRJNA251788	SAMN02840652	GCA_000732615	36.5	0.51
B. territorii CCUG65687	De Smet et al.2015[1]	*Burkholderiaterritorii*	66.6	6935	PRJNA563568	SAMN12697560	GCA_008802115	38	1.07

B. ubonensis LMG20358	Yabuuchi et al.2000(6)	*Burkholderia ubonensis*	67.2	6745	PRJEB33447	SAMEA5795714	GCA_902499185	52.9	0.38
B.semiarida CCRMBC74T	Velez et al. 2023(7)	*Burkholderia semiarida*	67.0	6635	PRJNA735651	SAMN19592388	GCA_029268935	40.7	0.63
B. sola CCRMBC51T	Velez et al. 2023(7)	*Burkholderia sola*	66.6	7381	PRJNA735651	SAMN19592387	GCA_029268985	39.3	0.98
B. seminalis LMG24067	Vanlaere et al.2008(3)	*Burkholderia seminalis*	67.1	7151	PRJEB33447	SAMEA5795711	GCA_902499165	40.7	0.55
B. metallica LMG24068	Vanlaere et al.2008(3)	*Burkholderia metallica*	67.1	6685	PRJEB33447	SAMEA5795708	GCA_902499065	39.9	0.58
B. pyrrocinia DSM10685	Vandamme et al.1997(8)	*Burkholderia pyrrocinia;*	66.5	6802	PRJNA283474	AMN03651233	GCA_001028665	42.7	1.17
B. ambifaria AMMD	Coenye et al.2001(9)	*Burkholderia ambifaria*	66.8	6610	PRJNA13490	SAMN02598309	GCA_000203915	41.4	0.86

B. stabilis ATCCBAA-67	Vandamme et al.2000(10)	*Burkholderia stabilis*	66.4	7425	PRJNA328254	SAMN05367054	GCA_001742165	38.6	1.21
B. contaminans LMG 23361	Depoortr et al.2020(11)	*Burkholderia contaminans*	65.9	7984	PRJNA335409	SAMN05441496	GCA_001758385	36.8	1.74
B. orbicola TAtl-371	Morales-Ruíz etal. 2022(12)	*Burkholderia orbicola; Burkholderia servocepacia*	67.0	6656	PRJEB16032	SAMN05443026	GCA_900100915	40.9	0.62
B. stagnalis CCUG65686	De Smet et al.2015(1)	*Burkholderia stagnalis*	67.0	7275	PRJNA563568	SAMN12697559	GCA_008802125	93.9	0.6

Table S8. IAA, siderophore, and nitrogen fixation related genes of *B. stagnalis* YJ-2.

	YJ-2 Gene ID	Function
	YJ-2_GM003017	amidase
	YJ-2_GM003116	aldehyde dehydrogenase
	YJ-2_GM004030	aldehyde dehydrogenase (NAD+)
	YJ-2_GM005295	monoamine oxidase
IAA	YJ-2_GM005308	monoamine oxidase
	YJ-2_GM005704	aldehyde dehydrogenase (NAD+)
	YJ-2_GM000027	aldehyde dehydrogenase (NAD+)
	YJ-2_GM000459	amidase
	YJ-2_GM000702	amidase
	YJ-2_GM004779	iron complex outermembrane recepter protein
	YJ-2_GM004865	ornibactin biosynthesis protein
	YJ-2_GM004866	ornibactin synthetase F
Ornibactin	YJ-2_GM004867	iron complex outermembrane recepter protein
	YJ-2_GM004868	L-ornithine N5-monooxygenase
	YJ-2_GM004869	ornibactin biosynthesis protein
	YJ-2_GM004870	orbJ ornibactin biosynthesis non-ribosomal peptide synthase

	YJ-2_GM004871	orbI ornibactin biosynthesis non-ribosomal peptide synthase
	YJ-2_GM004872	ornibactin biosynthesis ABC transport protein
	YJ-2_GM004874	iron complex transport system substrate-binding protein
	YJ-2_GM004875	ferric iron reductase protein FhuF
	YJ-2_GM004876	iron complex transport system permease protein
	YJ-2_GM004877	iron complex transport system ATP-binding protein
	YJ-2_GM004878	dioxygenase
	YJ-2_GM004879	MbtH protein
	YJ-2_GM004880	orbS extracytoplasmic-function sigma-70 factor
	YJ-2_GM006205	MbtH protein
Nitrogen fixation	YJ-2_GM003448	glutamine synthetase
	YJ-2_GM003575	nitrogen regulatory protein P-II 2
	YJ-2_GM004035	nitrogen regulatory protein P-II 1
	YJ-2_GM004349	nitrogen fixation protein NifU and related proteins
	YJ-2_GM005966	glutamate dehydrogenase (NAD(P)+)
	YJ-2_GM000921	glutamate dehydrogenase (NAD(P)+)
	YJ-2_GM006795	nitrogen fixation protein NifQ

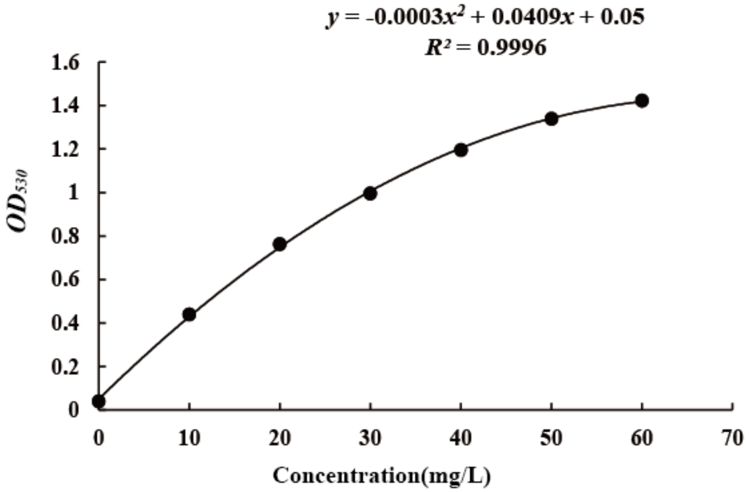

Figure S1. IAA standard curve.

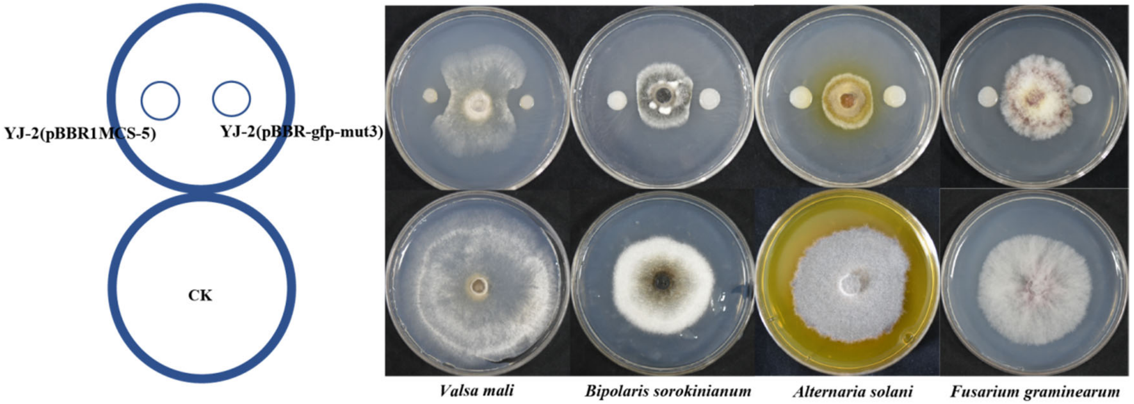

Figure S2. The antagonistic effects of *B. stagnalis* YJ-2 (pBBR-*gfp*-mut3) and *B. stagnalis* YJ-2 (pBBR1MCS-5) on plant pathogenic fungi.

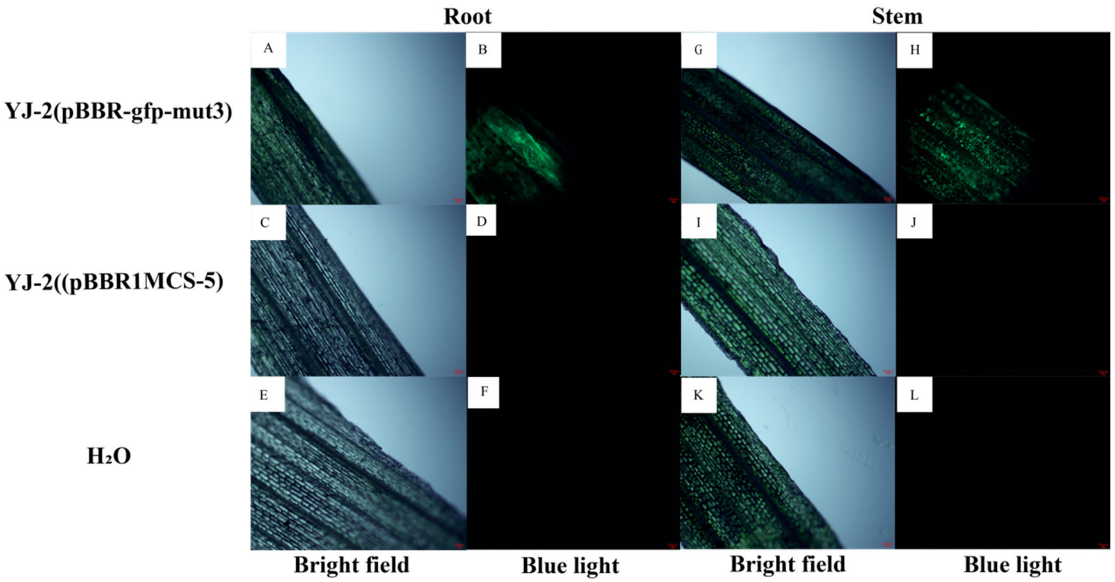

Figure S3. Colonization of *B. stagnalis* YJ-2 (pBBR-*gfp*-mut3) in wheat.

YJ-2 (pBBR-*gfp*-mut3), YJ-2 (pBBR1MCS-5) and water-treated wheat seedling root slices were observed for fluorescence under white light (A, C and E) and blue light (B, D and F) using a fluorescence microscope. YJ-2 (pBBR-*gfp*-mut3), YJ-2 (pBBR1MCS-5) and water-treated wheat seedling stem slices were observed for fluorescence under white light (G, I and K) and blue light (H, J, and L) using a fluorescence microscope. Bar = 50 μm.

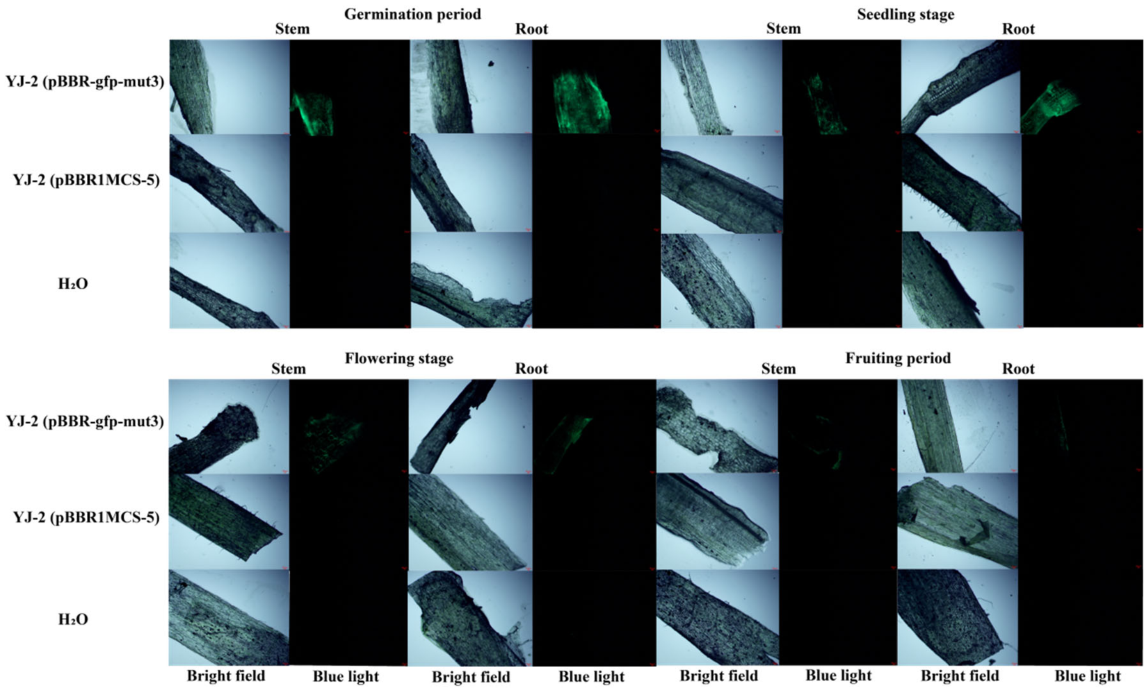

Figure S4. Colonization of *Burkholderia* sp. YJ-2 (pBBR-*gfp*-mut 3) during the tomato growth cycle.

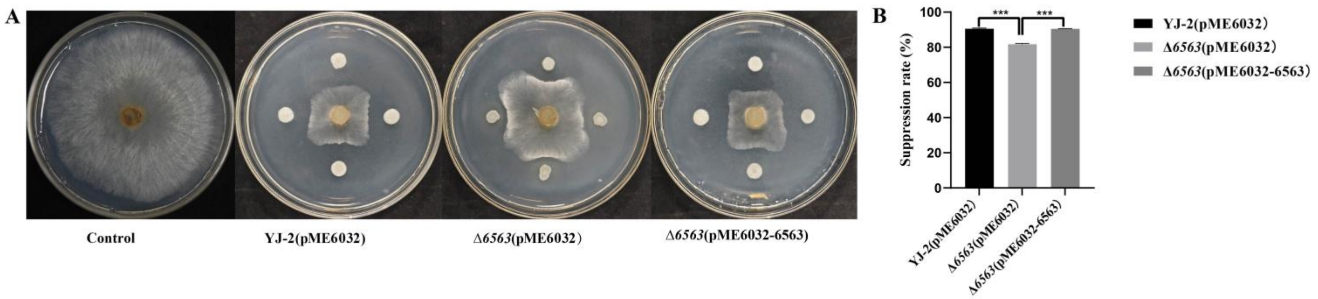

Figure S5. Effect of loss of YJ-2-GM006563 in *B. stagnalis* YJ-2 on antagonism of plant pathogenic fungi. (A) Antifungal effect of YJ-2(pME6032), *Δ6563*(pME6032), and *Δ6563*(pME6032-*6563*) on *V. mali*. (B) Inhibition rate of YJ-2(pME6032), *Δ6563*(pME6032), and *Δ6563*(pME6032-*6563*) against *V. mali*. All the data are representative of a minimum of three independent experiments. Error bars represent the standard deviations. ***p<0.001. n = 3

Reference

65 De Smet, B.; Mayo, M.; Peeters, Charlotte.; Zlosnik, J. E. A. ;Spilker, T.; Hird, T. J.; LiPuma. J. J.; Kidd, T. J.; Kaestli, M.; Ginther, J. L.; *et al*. Burkholderia stagnalis sp. nov. and Burkholderia territorii sp. nov., two novel Burkholderia cepacia complex species from environmental and human sources. *Int. J. Syst. Evol. Microbiol.* **2015**, *65*(Pt_7):2265-71.

78. Peeters, C.; Zlosnik, J.E.; Spilker, T.; Hird, T.J.; LiPuma, J.J.; Vandamme, P. Burkholderia pseudomultivorans sp. nov., a novel Burkholderia cepacia complex species from human respiratory samples and the rhizosphere. *Syst. Appl. Microbiol.* **2013**, *36*, 483–489, https://doi.org/10.1016/j.syapm.2013.06.003.

79. Vanlaere, E.; LiPuma, J.J.; Baldwin, A.; Henry, D.; De Brandt, E.; Mahenthiralingam, E.; Speert, D.; Dowson, C.; Vandamme, P. Burkholderia latens sp. nov., Burkholderia diffusa sp. nov., Burkholderia arboris sp. nov., Burkholderia seminalis sp. nov. and Burkholderia metallica sp. nov., novel species within the Burkholderia cepacia complex. *Int. J. Syst. Evol. Microbiol.* **2008**, *58*, 1580–1590, https://doi.org/10.1099/ijs.0.65634-0.

80. Bach, E.; Sant'Anna, F.H.; dos Passos, J.F.M.; Balsanelli, E.; de Baura, V.A.; Pedrosa, F.d.O.; de Souza, E.M.; Passaglia, L.M.P. Detection of misidentifications of species from the Burkholderia cepacia complex and description of a new member, the soil bacterium Burkholderia catarinensis sp. nov.. *Pathog. Dis.* **2017**, *75*, https://doi.org/10.1093/femspd/ftx076.

81. Kuan, S. O.; Yoong, K.A.; Learn, H.L.; Catherine, M. Y.; Yuen, L.C.; Sui, M. L.. Burkholderia paludis sp. nov., an Antibiotic-Siderophore Producing Novel Burkholderia cepacia Complex Species, Isolated from Malaysian Tropical Peat Swamp Soil. *Front Microbiol.* **2016**, *7* - 2016.

82. Yabuuchi,E.; Kawamura,Y.; Ezaki, T.; M Ikedo, Dejsirilert, S.; Fujiwara, N.; Naka,T.; Kobayashi, K.. Burkholderia uboniae Sp. Nov., l-Arabinose-Assimilating but Different from Burkholderia thailandensis and Burkholderia vietnamiensis. *Microbiol. Immunol.* **2000**, *44*(4):307-17.

83. Velez, L.S.; Aburjaile, F.F.; Farias, A.R.; Baia, A.D.; Oliveira, W.J.; Silva, A.M.; Benko-Iseppon, A.M.; Azevedo, V.; Brenig, B.; Ham, J.H.; et al. Burkholderia semiarida sp. nov. and Burkholderia sola sp. nov., two novel B. cepacia complex species causing onion sour skin. *Syst. Appl. Microbiol.* **2023**, *46*, 126415, https://doi.org/10.1016/j.syapm.2023.126415.

84. Vandamme, P.; Holmes, B.; Vancanneyt, M.; Coenye, T.; Hoste, B.; Coopman, R.; Revets, H.; Lauwers, S.; Gillis, M.; Kersters, K.; et al. Occurrence of Multiple

Genomovars of Burkholderia cepacia in Cystic Fibrosis Patients and Proposal of Burkholderia multivorans sp. nov.. *Int. J. Syst. Evol. Microbiol.* **1997**, *47*, 1188–1200, https://doi.org/10.1099/00207713-47-4-1188.

85. Coenye, T.; Mahenthiralingam, E.; Henry, D.; LiPuma, J.J.; Laevens, S.; Gillis, M.; Speert, D.P.; Vandamme, P. Burkholderia ambifaria sp. nov., a novel member of the Burkholderia cepacia complex including biocontrol and cystic fibrosis-related isolates.. *Int. J. Syst. Evol. Microbiol.* **2001**, *51*, 1481–1490, https://doi.org/10.1099/00207713-51-4-1481.
86. Vandamme, P.; Mahenthiralingam, E.; Holmes, B.; Coenye, T.; Hoste, B.; De Vos, P.; Henry,D.; Speert, D. P.. Identification and Population Structure ofBurkholderia stabilis sp. nov. (formerly Burkholderia cepacia Genomovar IV). *J. Clin. Microbiol.* **2000**;*38*(3):1042-7.
87. Depoorter E, De Canck E, Peeters C, Wieme AD, Cnockaert M, Zlosnik, J.E.A.; LiPuma, J. J.; Coenye, T.; Vandamme, P.. Burkholderia cepacia Complex Taxon K: Where to Split? *Front. Microbiol.* **2020**, *11* - 2020.
88. Morales-Ruíz, L.-M.; Rodríguez-Cisneros, M.; Kerber-Díaz, J.-C.; Rojas-Rojas, F.-U.; Ibarra, J.A.; Santos, P.E.-D.L. Burkholderia orbicola sp. nov., a novel species within the Burkholderia cepacia complex. *Arch. Microbiol.* **2022**, *204*, 1–9, https://doi.org/10.1007/s00203-022-02778-0.
